# Ku Regulates the Non-Homologous End Joining Pathway Choice of DNA Double-Strand Break Repair in Human Somatic Cells

**DOI:** 10.1371/journal.pgen.1000855

**Published:** 2010-02-26

**Authors:** Farjana Fattah, Eu Han Lee, Natalie Weisensel, Yongbao Wang, Natalie Lichter, Eric A. Hendrickson

**Affiliations:** Department of Biochemistry, Molecular Biology, and Biophysics, University of Minnesota Medical School, Minneapolis, Minnesota, United States of America; The Hospital for Sick Children and University of Toronto, Canada

## Abstract

The repair of DNA double-strand breaks (DSBs) is critical for the maintenance of genomic integrity and viability for all organisms. Mammals have evolved at least two genetically discrete ways to mediate DNA DSB repair: homologous recombination (HR) and non-homologous end joining (NHEJ). In mammalian cells, most DSBs are preferentially repaired by NHEJ. Recent work has demonstrated that NHEJ consists of at least two sub-pathways—the main Ku heterodimer-dependent or “classic” NHEJ (C-NHEJ) pathway and an “alternative” NHEJ (A-NHEJ) pathway, which usually generates microhomology-mediated signatures at repair junctions. In our study, recombinant adeno-associated virus knockout vectors were utilized to construct a series of isogenic human somatic cell lines deficient in the core C-NHEJ factors (Ku, DNA-PK_cs_, XLF, and LIGIV), and the resulting cell lines were characterized for their ability to carry out DNA DSB repair. The absence of DNA-PK_cs_, XLF, or LIGIV resulted in cell lines that were profoundly impaired in DNA DSB repair activity. Unexpectedly, Ku86-null cells showed wild-type levels of DNA DSB repair activity that was dominated by microhomology joining events indicative of A-NHEJ. Importantly, A-NHEJ DNA DSB repair activity could also be efficiently de-repressed in LIGIV-null and DNA-PK_cs_-null cells by subsequently reducing the level of Ku70. These studies demonstrate that in human cells C-NHEJ is the major DNA DSB repair pathway and they show that Ku is the critical C-NHEJ factor that regulates DNA NHEJ DSB pathway choice.

## Author Summary

Humans utilize at least two major pathways to repair DNA double-strand breaks (DSBs): homologous recombination (HR) and non-homologous end joining (NHEJ), and there are at least two genetically discrete sub-pathways of NHEJ: classical-NHEJ (C-NHEJ) and alternative-NHEJ (A-NHEJ). Since the products generated by each of these three repair (sub)pathways differ substantially from one another, it is biologically critical that certain DSBs are repaired by certain DSB repair pathways. How this pathway choice is made in human cells was unclear. In this study, knockout human cell lines that are defective in core C-NHEJ factors were generated. These cell lines are by-and-large extremely deficient in DSB repair, proving that C-NHEJ is the major DSB repair pathway in human cells. Unexpectedly, cell lines reduced for the C-NHEJ factors Ku70 or Ku86, carried out proficient DSB repair because of hyperactive A-NHEJ. In published work we have also demonstrated that Ku suppresses HR throughout the genome and at telomeres. Collectively, these data imply that Ku ensures that C-NHEJ is the major DSB repair pathway by two mechanisms: i) enabling C-NHEJ and ii) by actively suppressing HR and A-NHEJ. Thus, Ku is the critical regulator of pathway choice in human somatic cells.

## Introduction

One of the most harmful lesions a cell can encounter is a DNA double-strand break (DSB). In all organisms, efficient repair of these DSBs is critical for the maintenance of genomic integrity and viability [1]. Unfortunately, DSBs are frequently generated endogenously during normal cellular processes such as DNA replication, lymphoid V(D)J or class-switch recombination and are induced exogenously by the exposure to a variety of genotoxic agents such as ionizing radiation or chemotherapeutics [2]. Cells have conspired to meet this demand on their genetic material with the evolution of two mechanistically distinct pathways to repair DSBs: homologous recombination (HR), which takes advantage of either a homologous chromosome or a sister chromatid to join the broken DNA ends [3] and non-homologous end joining (NHEJ), a process that directly joins the DSB with little or no sequence homology between the broken ends [2]. In bacteria and lower eukaryotes, HR dominates the DNA DSB repair events whereas in higher eukaryotes, and especially in mammals, NHEJ is the preferred pathway for DNA DSB repair. NHEJ consists of at least two genetically and biochemically distinct sub-pathways: a main—“classic”—end-joining pathway (C-NHEJ) and one interchangeably referred to as microhomology-mediated end joining (MMEJ) [4], alternative NHEJ (A-NHEJ), or backup NHEJ (B-NHEJ) [5],[6] (hereafter referred to as A-NHEJ). C-NHEJ, while by no means precise, results in minimal DNA end processing, whereas A-NHEJ mechanistically results in deletions per force that are often accompanied by microhomology at the repair junction {[7],[8]; reviewed by [6],[9]}.

There are at least seven proteins required for C-NHEJ: Ku70, Ku86, the DNA dependent protein kinase catalytic subunit (DNA-PK_cs_), Artemis, X-ray cross complementing 4 (XRCC4), XRCC4-like factor (XLF) and DNA ligase IV (LIGIV) {reviewed by [10]}. The basic mechanism of C-NHEJ has been worked out in great detail. Ku70 and Ku86 form a heterodimer (Ku) that contains an internal cavity, which Ku uses to bind to and encircle broken DNA ends [11]. Ku, besides protecting DNA ends from exonucleolytic attack, also recruits DNA-PK_cs_, a phosphoinositol-3-like family serine/threonine protein kinase [12]. Together, Ku70, Ku86 and DNA-PK_cs_ form the DNA-dependent protein kinase complex (DNA-PK) and the assembly of this trimeric complex on the ends of double-stranded DNA activates the kinase activity of DNA-PK_cs_. DNA-PK_cs_, in turn, phosphorylates and activates the nuclease Artemis, which facilitates “cleaning up” of the ends. As a final step, ligation of the broken ends is catalyzed by the trimeric LIGIV complex, which consists of the catalytic core, DNA LIGIV, and its two accessory factors, XLF and XRCC4.

In contrast to C-NHEJ, the mechanism, the regulation and the factors involved in A-NHEJ remain elusive. Mechanistically, it is believed that during A-NHEJ both broken ends are resected 5′-to-3′ on one strand to generate 3′-single-stranded overhangs containing regions of microhomology (generally a few nucleotides), which are then used to mediate the repair event. Because of this reaction pathway, deletion of the sequences between the microhomologies occurs as does deletion of one of the blocks of (micro)homology. Moreover, the remaining block of microhomology always resides at the precise site of repair and can be used as a landmark to define such repair events [6],[9].

A-NHEJ was not thought to be a very robust nor particularly important DSB repair pathway because it could usually only be detected in the absence of C-NHEJ. Indeed, one of the first descriptions of A-NHEJ came with the observation that the few NHEJ DSB repair events that could be detected in Ku86-deficient budding yeast occurred between short direct repeats [7]. Since then, there have similar reports in fission yeast [13], frogs [14] and several mammalian systems [15]–[22] including humans [23]. The significance of—and parallel interest in—A-NHEJ increased with the demonstration that A-NHEJ could substitute at reasonable levels for C-NHEJ during DNA DSB repair events in murine lymphoid class switch recombination [24],[25] and during certain types of aberrant V(D)J recombination reactions [26],[27]. Moreover, A-NHEJ has been implicated in the generation of large deletions and other genomic rearrangements in murine cells [28]–[30]. Similarly, microhomology has been found at the recombination junctions of radiation-induced genomic rearrangements [31],[32] implying that even radiation-induced DSBs can be repaired by A-NHEJ. Lastly, microhomologies are frequently detected at breakpoints for chromosomal deletions and translocations in human cancer cells [33],[34]. These observations have propelled many laboratories to identify the factors required for A-NHEJ. These studies have implicated poly (ADP-ribose) polymerase-1 (PARP-1), X-ray cross complementing 1 (XRCC1), DNA ligase III (LIGIII), polynucleotide kinase (PNK), Flap endonuclease 1 (Fen-1) [5], [14], [35]–[37] and most recently the Mre11∶Rad50∶Nbs1 (MRN) complex (reviewed by [38]) but it is clear that additional factors await identification.

One of the most compelling questions in the DSB repair field is how pathway choice is determined. That is, once a chromosome breaks, how does the cell determine whether HR, C-NHEJ or A-NHEJ will mediate its repair? Since each of these repair pathways generates a discretely distinct product, the answer to this question is biologically important. Several laboratories have suggested that the relative abundance of factors, binding affinities for DNA ends, cell type specificity and/or cell cycle phases may impact upon this decision {reviewed in [39]}. These issues are complicated even more in human somatic cells where the impact of loss-of-function mutations on some of the C-NHEJ genes has distinctly different phenotypes than are observed in other mammals. In particular, Ku70 and Ku86 have evolved an essential telomere maintenance function that does not seem to be evident in any other mammalian studied to date [40]–[43]. Interestingly, Ku seems to exert this function by repressing the HR-mediated disassembly of telomeres [44] suggesting that pathway choice is critical for naturally occurring double-stranded DNA ends as well as broken ones.

To begin to experimentally address some of these issues we have generated a series of human somatic cell lines genetically engineered using recombinant adeno-associated virus (rAAV)-mediated gene targeting [45]–[47] to contain reduced levels of the C-NHEJ factors Ku70, Ku86, DNA-PK_cs_, XLF and LIGIV. We hypothesized that in the presence of reduced or no C-NHEJ activity the frequency and regulation of A-NHEJ in human cells could be assessed. To this end we utilized two *in vivo* plasmid assays that have been employed to study end joining in mammalian cells [26],[37],[48] to demonstrate that null mutations in DNA-PK_cs_, XLF or LIGIV resulted in a severe reduction in the frequency of productive DNA DSB repair. The small number of repair events that did occur in these null cell lines were hallmarked by the heavy usage of microhomology. Thus, these studies confirmed that C-NHEJ is the dominant NHEJ pathway operative inside human somatic cells and that in its absence small amounts of A-NHEJ can be detected. Very surprisingly, and in stark contrast to the results with DNA-PK_cs_, XLF and LIGIV-null cell lines, DNA DSB repair activity was actually slightly elevated in Ku86 conditionally-null cell lines. These repair events appeared, once again, to be heavily biased towards microhomology-mediated repair. This result suggested that Ku86 actively suppresses A-NHEJ in human somatic cells. This hypothesis was confirmed by using molecular and genetic approaches to reduce the levels of Ku70 in cell lines that were null for either DNA-PK_cs_ or LIGIV and which resulted in cell lines that had regained a DNA DSB repair activity that was mediated by microhomology. Together, these studies demonstrate that Ku (Ku70 and Ku86) is the critical regulator of pathway choice in human somatic cells.

## Results

### Strategy and cell lines

**Figure 1:**
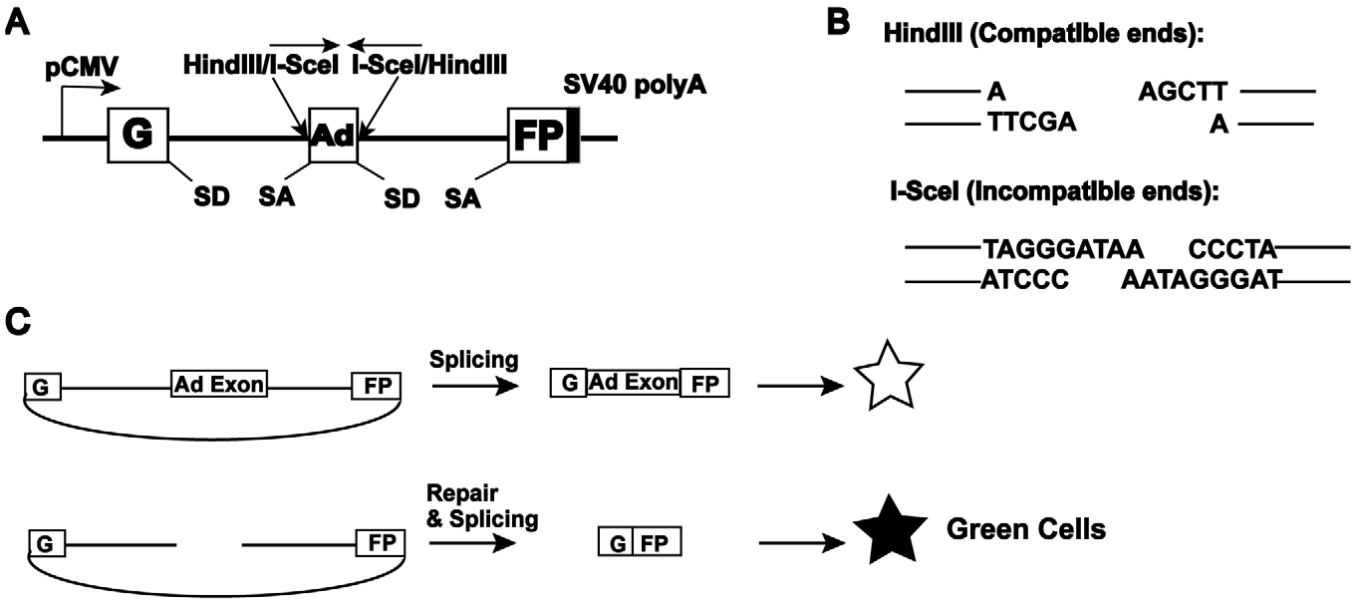
Reporter substrate for analysis of NHEJ. A cartoon of the reporter construct (pEGFP-Pem1-Ad2). (A) The construct is essentially a GFP cassette whose expression is driven by CMV promoter and terminated by the SV40 polyA sequence. “G” is separated from “FP” by a 2.4 kb intron containing an exon (Ad) from adenovirus that is flanked by *HindIII* and *I-SceI* restriction sites. Splice donor (SD) and splice acceptor (SA) sites are shown. (B) Restriction sites used to introduce DSBs. Digestion with *HindIII* generates compatible cohesive ends. Because *I-SceI* has a nonpalindromic 18-bp recognition site, cleavage of the two inverted *I-SceI* sites generates incompatible ends. (C) Due to the presence of the Ad-exon into the middle of the Pem1 intron, the Ad exon is efficiently spliced into the middle of the GFP ORF, inactivating the GFP activity and thus making the starting substrate GFP negative. Both sides of the Ad exon have *HindIII/I-SceI* restriction sites. Cleavage with either of these endonucleases removes the Ad exon and upon successful intracellular plasmid circularization GFP expression is restored and can be quantitated by flow cytometry.

To elucidate the role of C-NHEJ factors in DNA DSB repair, we made use of an extrachromosomal reporter assay system {[37],[48]; Figure 1}. This assay permits, in addition to the generation of defined DSBs, a detailed follow up of the repair of the reporter plasmid. In this assay, end joining is measured by the reconstitution of green fluorescent protein (GFP) expression [48]. The reporter pEGFP-Pem1-Ad2 consists of the GFP gene engineered such that it is interrupted by a 2.4 kb intron derived from the rat Pem1 gene (Figure 1A). An exon derived from adenovirus (Ad2) has been introduced into the middle of the intron and it is flanked on both sides by *HindIII* and *I-SceI* restriction enzyme recognition sequences. In the unmodified configuration, GFP is not expressed because the Ad2 exon is efficiently incorporated into the GFP mRNA (Figure 1C). Digestion of the plasmid either with *HindIII* or *I-SceI* at the flanking sites generates a linear plasmid lacking the adenoviral exon with either compatible 5′-overhanging cohesive ends or incompatible ends, respectively (Figure 1B). The *HindIII* sites are arranged such that cohesive 4-bp overlapping ends are generated, whereas the *I-SceI* sites are arranged in an inverted orientation, which demands that some sort of processing must occur before the ends can be rejoined. Thus, the impact of loss-of-function NHEJ gene mutations on these aspects of end joining can be individually assessed. Un-digested or partially digested plasmids, because of the retention of the Ad2 exon, generate a product unable to express GFP. Due to the buffering capacity of the intron, end joining by the cellular repair apparatus of transfected, linearized plasmid usually re-constitutes GFP expression, even when extensive additions or deletions of nucleotides have occurred (Figure 1C). As a result, a wide spectrum of end joining events can be detected and quantitated by FACS (fluorescently activated cell sorting). As a transfection control, cells are always co-transfected with a pCherry expression plasmid and the data are expressed as the percentage of cherry-positive cells that are also green-positive. Lastly, pEGFP-Pem1-Ad2 contains a bacterial origin of replication and an antibiotic resistance gene permitting the plasmids to be recovered from human cells and rescued in *E. coli*. Consequently, the structure of the repair junctions, which provides mechanistic insight into the type of repair that was utilized, can be identified by DNA sequencing.

This assay system was used to interrogate a series of isogenic human HCT116 cell lines. The derivative cell lines were engineered using rAAV gene targeting [46],[49] to be reduced or deficient in the expression of most of the C-NHEJ factors, namely: Ku70 [42],[50], Ku86 [40],[44], DNA-PK_cs_ [51], XLF (Fattah *et al*., unpublished) or LIGIV (Oh *et al*., unpublished). HCT116 is a human adenocarcinoma somatic tissue culture cell line that is mismatch repair defective [52],[53] and it is important to note that defects in mismatch repair genes have been implicated in affecting both HR- and NHEJ-mediated DSB repair efficacy {[54],[55] reviewed by [56]}. Moreover, it is also relevant that the cell line contains reduced levels of Mre11, Rad50 and Nbs1 [57],[58], a trimeric complex of proteins that have documented roles in HR and which have recently also been implicated in both C- and A-NHEJ {[59]–[61]; reviewed in [38]}. Despite these deficiencies, HCT116 is only slightly reduced for general NHEJ activity [62]. In addition, it is diploid, has a stable karyotype, exhibits normal DNA damage checkpoints and is wild-type for most of the other major DNA DSB repair genes [47]. These facts, combined with the previous extensive use of HCT116 in gene targeting studies [47] recommended this cell line for these analyses.

### LIGIV is the only C-NHEJ gene that is haploinsufficient for plasmid DNA end joining *in vivo*

**Figure 2:**
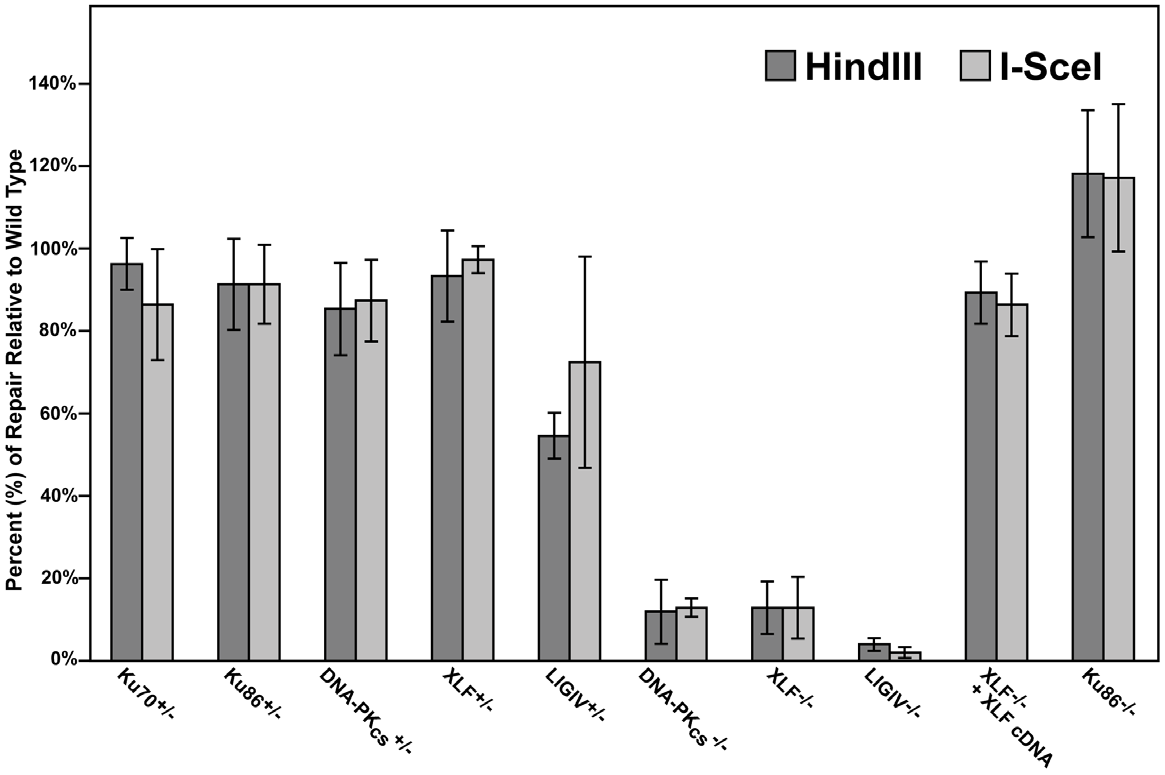
The impact of C-NHEJ mutations on end joining. Four independent experiments comparable to those depicted in Figure S1 and Figure 4 were performed and the average percent repair relative to wild-type is shown with the standard deviation.

When *HindIII-* or *I-SceI-*linearized pEGFP-Pem1-Ad2 plasmid was introduced into the parental HCT116 cell line, intracellular circularization allowing GFP expression could easily be detected and quantitated by flow cytometry (Figure S1). When the same experiment was carried out with the C-NHEJ heterozygous cell lines, significant repair activity was always observed (Figure S1). Averaged over four experiments, the Ku70, Ku86, DNA-PK_cs_ and XLF heterozygous cell lines showed only a slightly reduced ability to repair this DSB that was not significantly different from wild-type (Figure 2). In contrast, the LIGIV^+/−^ cell line possessed only ∼65% the repair capacity of the parental cell line and was reproducibly haploinsufficient (Figure S1 and Figure 2). In no case was a significant difference in repair frequency between the repair of *HindIII-* or *I-SceI-*plasmid observed (Figure 2). While the FACS analysis (Figure S1 and Figure 2) measured repair frequency, the repaired plasmids could also be analyzed molecularly. In particular, the *HindIII*-cleaved substrate contained 4 bp compatible overhangs that essentially constituted a stretch of microhomology (Figure 1B). If these sequences are used to mediate the repair event, they generate a slightly smaller plasmid that now contains a single *HindIII* restriction enzyme recognition site where formerly there were two. Consequently, the recovered, repaired *HindIII-*linearized pEGFP-Pem1-Ad2 plasmids were re-digested with *HindIII* before gel electrophoresis and the frequency of plasmids that had reconstituted a single *HindIII* site (“perfect joins”) was determined. Ku70^+/−^, Ku86^+/−^ (Table S1), DNA-PK_cs_^+/−^ (Table S3), XLF^+/−^ (Table S5) and LIGIV^+/−^ (Table S7) cells perfectly rejoined an average of ∼37% of the substrates, which was slightly higher than the 23% perfect rejoining observed in wild-type cells (summarized in Figure S2 and Figure 5B). Thus, even though the repair activity was not substantially affected by the loss of one allele of any of the C-NHEJ genes tested, the repair profiles shifted towards microhomology-mediated joining. In several instances, perfectly joined plasmids (as assessed by restriction digest and gel electrophoresis) were sequenced and without exception the existence of the expected single *HindIII* site was confirmed (data not shown). Lastly, plasmids for those *HindIII*-linearized plasmids that did not perfectly rejoin and 30 plasmids for all of the *I-SceI*-linearized plasmids (which can not perfectly rejoin) were sequenced. This analysis demonstrated that the size of the accompanying deletions (Figure S4, S5), the frequency of microhomology usage, and the frequency of insertions was, with a few interesting exceptions (see the Discussion), comparable to that observed in wild-type cells (Tables S2, S4, S6, S8 and Figure S2, S3). From these experiments, we concluded that the reduction by one allele of most C-NHEJ factors is generally aphenotypic for DNA end joining whereas LIGIV is more haploinsufficient, implying that LIGIV may be a limiting C-NHEJ factor in human somatic cells. This latter conclusion supports a prediction made based on the behavior of purified ligases *in vitro* [63].

### The absence of DNA-PK_cs_, XLF, and LIGIV greatly reduces DNA repair activity

**Figure 3:**
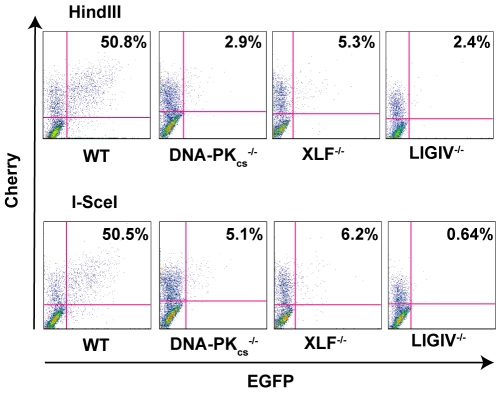
The loss of C-NHEJ greatly reduces end joining. The indicated cell lines were transfected with *HindIII*- (Top panels) or I*-SceI*- (Bottom panels) linearized pEGFP-Pem1-Ad2 plasmid together with a pCherry plasmid. The cells were analyzed by FACS 24 hr post transfection. The number of cells that were doubly EGFP (horizontal) and pCherry (vertical) positive versus the number that were pCherry positive was determined. For a given experiment the data are shown as percent repair in the upper right-hand corner of each plot.

In rodents, cells deficient in any of the C-NHEJ components are generally very deficient in joining virtually all types of DSBs [1]. To test whether C-NHEJ-deficient human cells are also impaired in end joining we repeated the above experiment in DNA-PK_cs_-, XLF- and LIGIV-null cell lines. In all three cases, the frequency of end joining was greatly reduced (Figure 3). On average, DNA-PK_cs_- and XLF-null cell lines were diminished by an order-of-magnitude and showed only about 10% the repair activity observed in the parental cell line (Figure 2). The LIGIV-null cell line was always the most profoundly affected cell line and performed end-joining only a few percent above background. The fact that XLF-null cells were reproducibly more active than the LIGIV-null cells is consistent with XLF playing an important, but not essential, role in DNA DSB ligation. We next attempted—as a proof-of-principle—to functionally rescue the XLF-null line to confirm that the loss of end-joining activity was due specifically to the respective targeted knockout mutations in these cell lines. A XLF cDNA was stably introduced via retroviral infection into the XLF-null cell line and a subclone expressing wild-type levels of XLF protein was isolated (data not shown). This cell line showed an end-joining activity that was 90% of wild-type (Figure 2) directly demonstrating that the absence of XLF was responsible for the phenotype of the null cells. In conclusion, these experiments demonstrated that C-NHEJ is the major NHEJ repair pathway in human somatic cells and in its absence only low (albeit detectable—see below) levels of end joining can occur.

### The absence of Ku changes the repair profile, but not the repair activity

**Figure 4:**
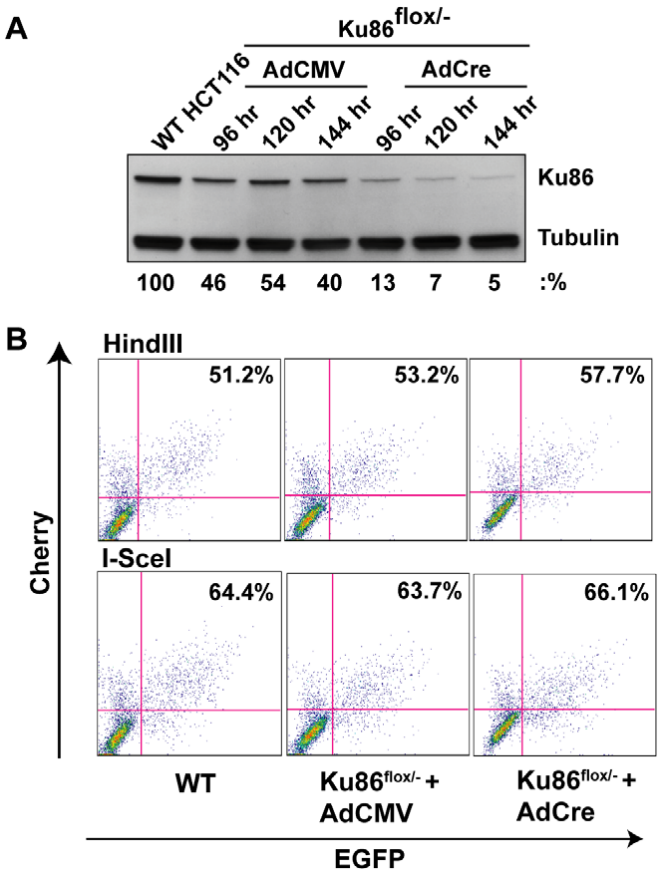
Ku86-null cells show wild-type levels of end joining activity. (A) Western blot analysis shows that the expression of Cre (AdCre) in Ku86^flox/−^ cells results in the reduction of Ku86 expression. AdCMV is a negative control adenoviral vector. (B) the indicated cell lines were transfected with either HindIII- (Top panels) or I-SceI- (bottom panels) linearized pEGFP-Pem1-Ad2 plasmid. All symbols are as in Figure 3.

Primates are unique in that, in contrast to every other species examined to date, the Ku genes have evolved to become essential [40],[42],[44], due to their ability to suppress lethal HR-mediated telomere recombination [43]. Consequently, human cell lines that are null for either Ku70 [42] or Ku86 [40] are not viable. We have, however, constructed a “conditionally null” (Ku86^flox/−^) cell line for Ku86. This cell line has been engineered through rAAV gene targeting technology to contain only a singly functional “floxed” allele of Ku86 [44]. In the presence of the Cre recombinase, the floxed allele is excised and the cells become null for Ku expression. Importantly, the loss of Ku86 is essentially complete in 3 to 4 days and although the cells will ultimately succumb, they generally don't do so for approximately 2 weeks [44]. Thus, the day 4 to day 14 window was used to assess the ability of the cells to perform end joining. Consequently, Ku86^flox/−^ cells were either infected with a control adenovirus (AdCMV) or an adenovirus expressing Cre (AdCre). At 4, 5 and 6 days post-infection, a portion of the cells were processed for Western analysis, which confirmed that the levels of Ku86 protein were greatly diminished in the AdCre-treated cells compared to the control AdCMV-treated cells (Figure 4A). The levels of Ku86 never go to zero because a minor portion of the cells are either not productively infected with the adenoviral vector and/or they do not efficiently undergo Cre-mediated recombination [44]. At 120 hr post adenovirus infection, the cells were transfected with pEGFP-Pem1-Ad2 and 24 hr later the cells were analyzed by FACS analysis. Very unexpectedly, Ku86^flox/−^ +AdCre cells performed end joining at a wild-type frequency (Figure 4B). Indeed, in four independent experiments the “Ku86-null” cells reproducibly seemed to have even slightly higher levels of end joining activity than wild-type cells, regardless of whether *HindIII-* or *I-SceI-*linearized substrates were used (Figure 2).

**Figure 5:**
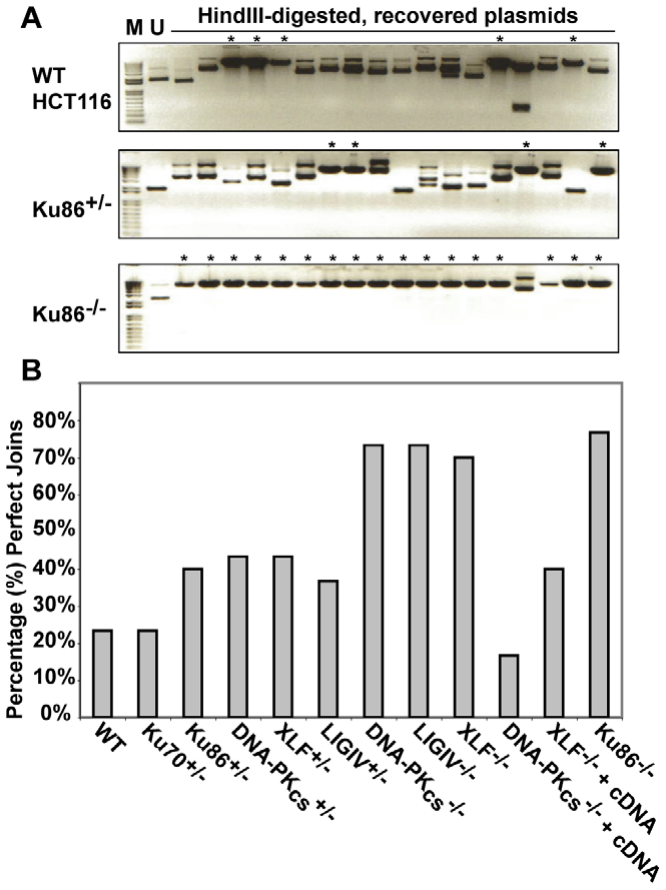
The absence of Ku86 results in predominately microhomology-based end joining. (A) *HindIII*-linearized pEGFP-Pem1-Ad2 plasmids were recovered from either WT HCT116, Ku86^+/−^ or Ku86^−/−^ cells, propagated through *E. coli* and then analyzed for retention of a single *HindIII* restriction site (“perfect rejoining”) by *HindIII* restriction enzyme digestion analysis. The asterisks indicate those plasmids where perfect rejoining occurred. (B) The results of four or more experiments similar to those depicted in (A) were combined and summarized. *N.B*. The re-expression of a WT DNA-PK_cs_ or XLF cDNA (+cDNA) in their respective null cell lines reduced the frequency of perfect rejoining back to WT levels, confirming the specificity of the effect.

We were perplexed by this result until we considered the possibility that although the frequency of end-joining was not altered in Ku86-null cells the repair profile might be. To experimentally test this hypothesis, the repaired *HindIII*-cleaved pEGFP-Pem1-Ad2 substrate plasmids were recovered from Ku86-null cells and analyzed by agarose gel electrophoresis following *HindIII* re-digestion for perfect rejoining. In the parental and heterozygous cell lines this type of repair event was observed in about 30% of the repaired plasmids (asterisked lanes in Figure 5A and 5B). In striking contrast, ∼80% of all the plasmids recovered from Ku86-null cells had reconstituted a single *HindIII* site (Figure 5A and 5B). Thus, while the overall repair frequency in Ku86-null cells was not significantly different from wild-type cells, the repair profile was heavily shifted to one that utilized more microhomology.

### Microhomology-mediated end joining also dominates in the absence of DNA-PK_cs_, XLF, and LIGIV

Although end joining was greatly reduced in DNA-PK_cs_-, XLF- and LIGIV-null cell lines (Figure 3), it was not zero. Given the above results with Ku86-null cells, we next tested whether the residual repair in these other C-NHEJ-null cell lines was also heavily biased towards microhomology. Indeed, although there were far fewer repair events in these three cell lines in comparison to Ku86-null cells, they were nonetheless predominantly (70% to 80%) mediated by microhomology (Figure 5B). Thus, DNA-PK_cs_-, XLF- and LIGIV-null cell lines had an identical repair profile to Ku86-null cells (Figure 5B), but carried out only 1% to 10% as many repair events as Ku86-null cells.

### A-NHEJ is negatively regulated by Ku in human somatic cells

The above results suggested that Ku normally actively suppresses A-NHEJ. In Ku's presence, even when C-NHEJ is inactivated by mutations in DNA-PK_cs_, XLF or LIGIV, A-NHEJ is apparently still strongly suppressed (Figure 3). In contrast, in Ku's absence, A-NHEJ is “unleashed” and rescues the repair activity of the cells (Figure 4). Interestingly, there is precedent for this model in the literature. Thus, mouse cell lines deficient for Ku86 repaired *I-SceI*-induced DSBs with a frequency similar to that of wild-type cells but with a repair profile that was biased towards microhomology [17]–[21],[64]. Moreover, the ionizing radiation sensitivity of LIGIV-deficient chicken DT40 cells can be rescued by the deletion of Ku70 [65]. Perhaps most impressively, LIGIV deficiency in the mouse results in embryonic lethality and this can be rescued by the deletion of Ku86 [66]. Although, repair profiles were not assessed in the latter two studies, they are consistent with the absence of Ku de-repressing A-NHEJ to the point where the phenotypes could be rescued.

**Figure 6:**
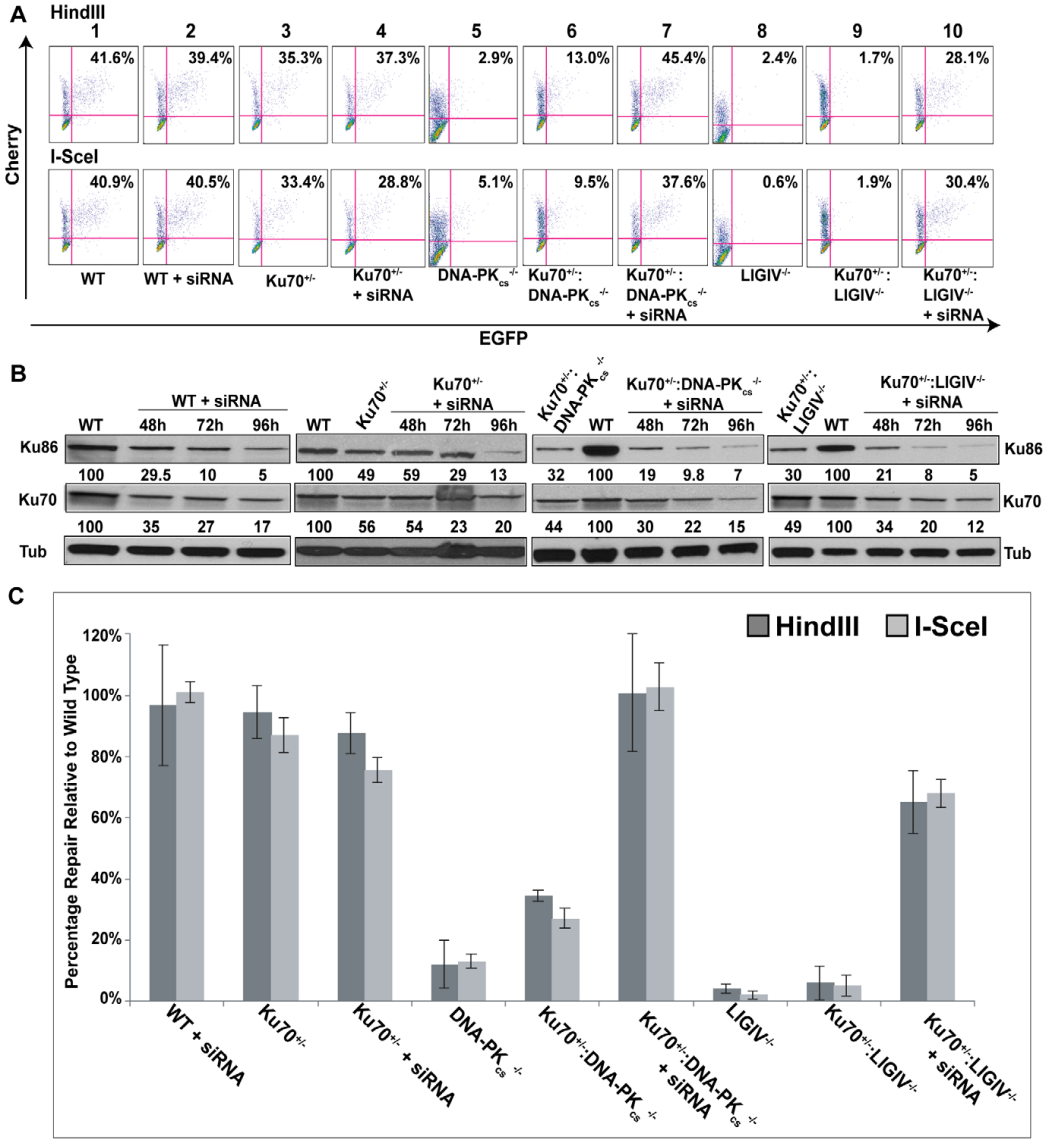
The reduction of Ku results in elevated levels of end joining in C-NHEJ mutant cell lines. (A) FACS profiles using the pEGFP-Pem1-Ad2 reporter substrate are shown for the indicated cell lines. The profiles for the Top and Bottom panels were generated using *HindIII*- and *I-SceI*-linearized plasmids, respectively. The percent of the substrate that was repaired is shown in the right-hand corner of each profile. (B) Western blot analyses demonstrate a reduction in Ku protein levels. Western blots for extracts derived from the indicated cell lines are shown using antibodies against either Ku86, Ku70 or (as a loading control) tubulin (Tub). Each of the blots was quantitated using a phosphoimager and the level of a particular Ku subunit relative to the amount expressed in the parental cell line is indicated below each blot. (C) Four independent experiments comparable to those depicted in (A) were performed and the average percent repair is shown with the standard deviation.

To investigate if this paradigm could be extended to human cells we directly tested whether the strong repair defects of DNA-PK_cs_- and the very severe defects of LIGIV-null cells could be rescued by reducing the amount of Ku in these cell lines. A combination of genetic and molecular approaches was utilized to achieve a significant knockdown of Ku, a highly abundant protein. Thus, rAAV gene targeting was first used to functionally inactivate one Ku70 allele in DNA-PK_cs_- and LigIV-null cell lines. DNA-PK_cs_^−/−^∶Ku70^+/−^ and LIGIV^−/−^∶Ku70^+/−^ cell lines have ∼50% the level of Ku70 protein compared to wild-type cells (Figure 6B; [42],[50]) and this reduction in Ku slightly rescued the repair deficiencies of either cell line (compare panel 5 with panel 6 and panel 8 with panel 9 in Figure 6A; Figure 6C). siRNA against Ku70 was then used to reduce the level of Ku protein to ∼5% of wild-type (+siRNA, Figure 6B). Impressively, DNA-PK_cs_^−/−^ and LIGIV^−/−^ cells showed wild-type and greatly enhanced, respectively, end-joining activity (compare panel 5 with panel 7 and panel 8 with panel 10 in Figure 6A; Figure 6C), directly demonstrating that a reduction in Ku can “reanimate” a cell that appears “dead” for DNA DSB repair. Importantly, the end joining occurring in these Ku-reduced cell lines was predominately microhomology mediated (Tables S3, S4, S7, S8). Moreover, these data provide a plausible molecular mechanistic explanation for the earlier genetic results obtained in chickens and mice.

### Microhomology-mediated A-NHEJ predominates in C-NHEJ deficient cells

**Figure 7:**
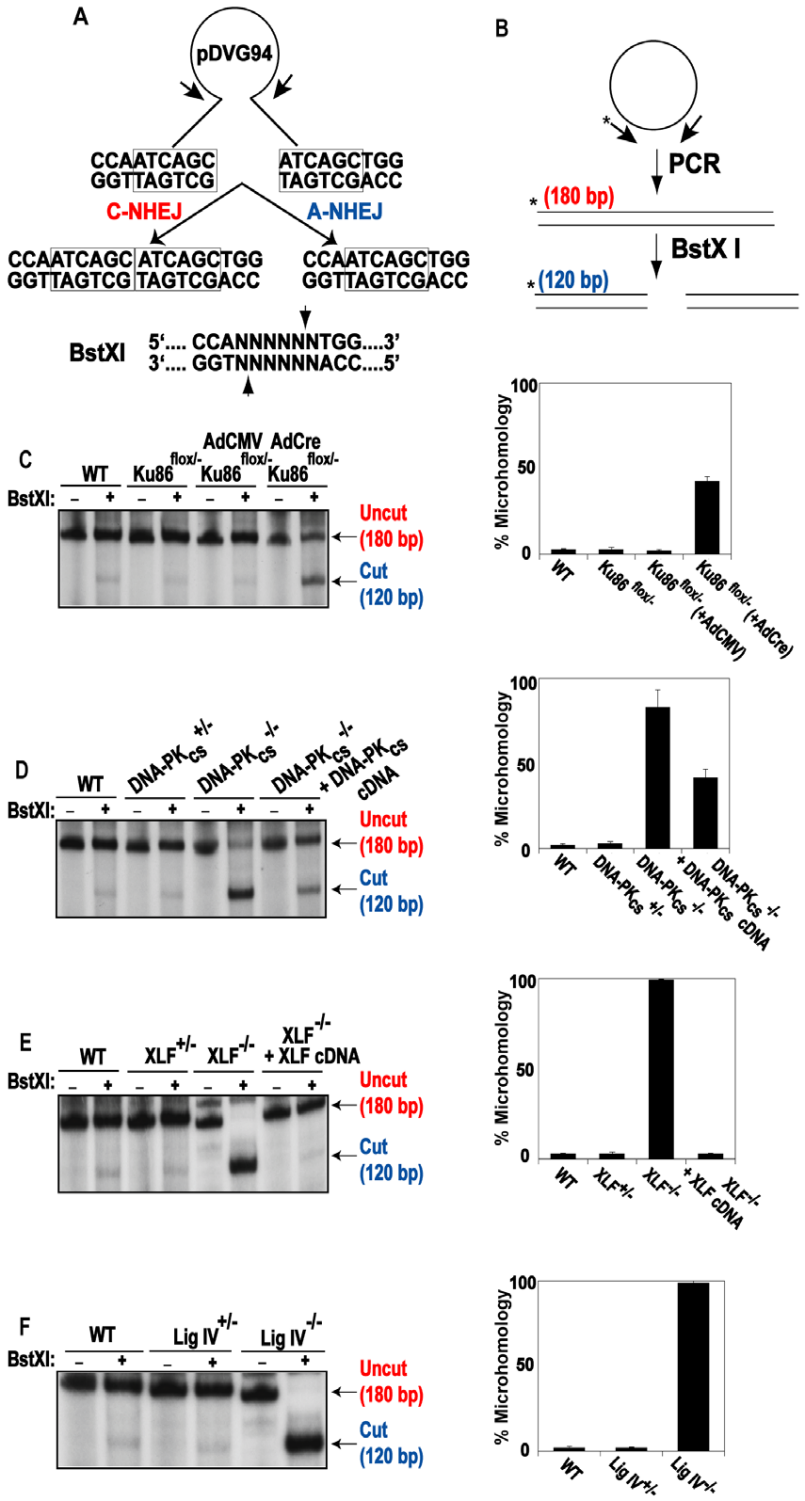
Independent confirmation of microhomology-mediated end joining in C-NHEJ mutant cell lines. (A) Reporter substrate biased for use by microhomology-directed NHEJ (A-NHEJ). The reporter has been designed such that cleavage with *Eco47III* and *EcoRV* results in a blunt-ended linear substrate with 6-bp direct repeats (boxes) at both ends. C-NHEJ joining will result in the retention of some of both repeats whereas A-NHEJ should generate a single repeat, which is a substrate for *BstXI*. This figure is excerpted from Verkaik *et al*., 2002, Eur. J. Immunol., 32∶701. (B) The experimental scheme for analysis of the plasmids recovered from transfected cells. The plasmids were subjected to PCR using one radiolabeled (asterisk) primer. The PCR products were then subjected to *BstXI* restriction enzyme digestion. (C–F) Left Panels: Autoradiograms of representative microhomology assays using the indicated cell lines. The size of the primary PCR product (180 bp) and the *BstXI* cleavage product (120 bp) are indicated. Right Panels: Three independent experiments similar to the ones shown on the left were quantitated with a phosphoimager and averaged.

To confirm and extend the above results, we utilized a reporter assay that is biased towards detecting A-NHEJ events. pDVG94 is designed such that the relative efficiency of C-NHEJ versus A-NHEJ events can be assessed [26],[67]. When pDVG94 is digested with *AfeI* and *EcoRV* it results in a blunt-ended linear substrate with a 6-bp repeat at both ends (Figure 7A). C-NHEJ can rejoin these ends and yield a wide variety of junctions but A-NHEJ almost exclusively generates a single product in which the 2 repeats have been reduced to 1, which simultaneously generates a novel *BstXI* restriction enzyme recognition site (Figure 7A). Thus, linearized pDVG94 plasmid was transfected into the mutant cell lines and 48 hr later repaired plasmids were recovered, purified and then used as substrates for PCR using a 5′-radiolabeled PCR primer (Figure 7B). The relative level of A-NHEJ is subsequently determined by quantification of the *BstXI*-digested PCR products where a 180 bp product represents the repaired plasmid and a cleaved 120 bp product is diagnostic of microhomology-mediated end joining (Figure 7B). The parental cell line, Ku86^flox/−^ and Ku86^flox/−^ infected with AdCMV cell lines carried out only a few percent of microhomology-mediated end joining in this assay (Figure 7C). In contrast, Ku86-null cells showed on average 45% microhomology use (Figure 7C). Although this assay cannot be used to determine the absolute frequency of the individual repair events, it confirmed that in the absence of Ku, microhomology-mediated events became easily detectable.

This phenotype was even more evident in the DNA-PK_cs_- (Figure 7D), XLF- (Figure 7E) and LIGIV- (Figure 7F) null cell lines. In the absence of one of these three factors, the frequency of microhomology-mediated end joining was virtually 100%. Importantly, the re-introduction of a wild-type DNA-PK_cs_ or XLF cDNA, into their respective null cell line, partially and completely, respectively, reverted the repair events to a C-NHEJ spectrum. Again, the degree of complementation was directly related to the degree of complementing protein expression achieved in these cell lines (data not shown).

These experiments demonstrated that in the presence of Ku, but in the absence of other C-NHEJ factors, that virtually all of the end joining in human cells is carried out by microhomology-mediated processes. In contrast, in the presence of the other C-NHEJ factors, but in the absence of Ku, some, but not all (see the Discussion), of the end joining occurs using microhomology.

### Ku protects DNA ends from degradation

In every metabolic reaction (*e.g*., DSB repair, V(D)J recombination, telomere maintenance, *etc*.) that Ku participates in, and in every organism that such reactions have been characterized, Ku's absence is marked by hyper-resection of the relevant DNA ends [11]. To determine if this aspect of Ku's absence is conserved in human cells extensive sequencing was carried out of pEGFP-Pem1-Ad2 plasmids recovered from wild type and Ku86-null cells. A significant increase in deletion size in Ku86-null cells compared to wild-type cells was observed. In wild-type cells the median deletion size was 595 bp whereas in Ku86-null cells it was 1158 bp for *HindIII*-linearized plasmids (Table S1 and Figure S4). This same trend was also observed in *I-SceI*-linearized plasmids as the median deletion size was 1097 bp in Ku86-null cells in comparison to 321 bp in wild type cells (Table S2 and Figure S5). When the same analysis was carried out for DNA-PK_cs_-null (Table S3, S4), XLF-null (Table S5, S6.) and LIGIV-null (Tables S7, S8) cell lines less degradation of the DNA ends compared to wild-type cells was observed (Figure S4, S5). In summary, the absence of Ku in human somatic cells carried with it a hyper-resection phenotype that was identical to that observed for Ku-dependent reactions in all other species.

### A-NHEJ in the absence of Ku in rodent cell lines

**Figure 8:**
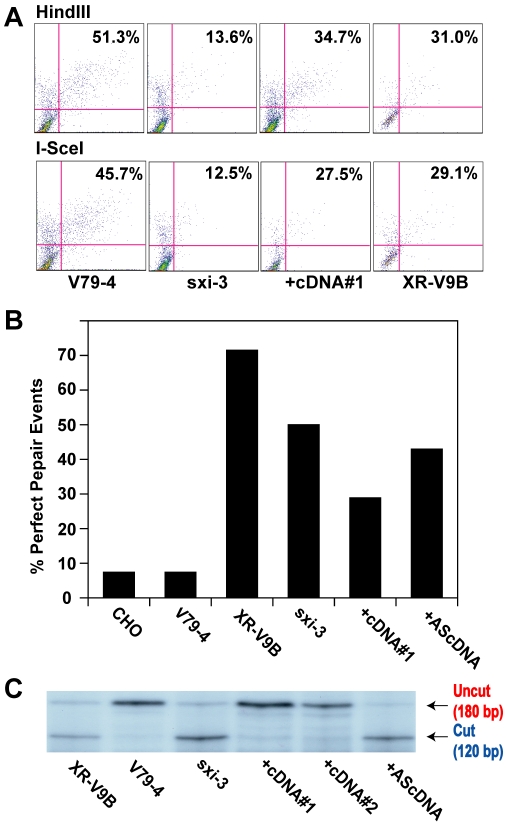
Microhomology-mediated end joining in hamster Ku86-null cell lines. (A) The indicated cell lines were transfected with *HindIII*- (Top panels) or *I-SceI*-(Bottom panels) linearized pEGFP-Pem1-Ad2 together with a supercoiled pCherry plasmid (to monitor transfection efficiency). The number in the top right corner corresponds to the percentage of cells that turned green after 24 hr as a percentage of the cells productively transfected. +cDNA#1 corresponds to a *sxi-3* cell line that has been stably complemented with a Ku86 cDNA. (B) *HindIII*-linearized pEGFP-Pem1-Ad2 plasmids were recovered from the indicated cell lines, propagated through *E. coli* and then analyzed for retention of a single *HindIII* restriction site (“perfect rejoining”) by *HindIII* restriction enzyme digestion analysis. (C) The indicated cell lines were transfected with the pDVG94 plasmid that had been linearized by *Eco47III* and *EcoRV* digestion. After 24 hr, the plasmids were recovered and then analyzed by PCR as described in Figure 7.

Given that the hyper-resection phenotype was conserved between human somatic cells and other mammals, we anticipated that the repair phenotypes we have described would also be conserved. To experimentally test this prediction, we utilized two well-characterized hamster cell lines that are defective in Ku86 expression, *XR-V9B* [68] and *sxi-3* [69]. The “parental” cell line from which these two mutant lines were isolated, is the hamster lung cell line, V79-4. Similar to HCT116 cells, the V79-4 cell line was able to convert about 50% of the pEGFP-Pem1-Ad2 linearized plasmid to circularized product regardless of whether it had been linearized with *HindIII* or *I-SceI* (Figure 8A). Unlike the human Ku86-null cell line however, the *sxi-3* mutant cell line was significantly reduced in its ability to repair, although the *XR-V9B* line had intermediate activity (Figure 8A). These results were very similar with a published report using another hamster cell line, *xrs-6* and its complemented control, where a 5-fold reduction in repair frequency was observed for the mutant line [37]. Thus, 3 independent hamster Ku86-null cell lines showed a deficit in their repair frequency, which was different from what we have observed in Ku-depleted human somatic cells. To investigate this in more detail, we recovered 14 originally *HindIII*-linearized plasmids from each of these cell lines and several additional control cell lines and determined the percentage of perfect joins in the products by attempting to re-cleave them with *HindIII*. Only a single perfectly rejoined plasmid (1/14 = 7%) was recovered from either V79-4 cells or the related “wild-type” Chinese hamster ovary (CHO) line (Figure 8B), consistent with C-NHEJ being the major repair pathway in “normal” hamster somatic cell lines as well. In contrast, both *XR-V9B* (10/14 = 71.4%) and the *sxi-3* (7/14 = 50%) cell lines showed heavy usage of microhomology-mediated repair events (Figure 8B). Derivative *sxi-3* cell lines, which express either a complementing cDNA (+cDNA#1) or a cDNA expressed in the anti-sense orientation (+AScDNA) have been described [70]. These cell lines showed a restoration of C-NHEJ (3/14 = 21.4%) and a lack of restoration (6/14 = 42.9%), respectively. To corroborate these results, the cell lines were also subjected to the independent repair assay using the linearized pDVG94 plasmid. In this instance, both *XR-V9B* and *sxi-3* again showed a much higher usage of A-NHEJ than the parental V79-4 cell line and this could be rescued in two independent cell lines containing a complementing cDNA, but could not be rescued in the cell line containing the cDNA expressed in the anti-sense orientation (Figure 8C). From these experiments, we conclude that although the loss-of-function of Ku86 phenotypes in human cells is mimicked in the Ku86-null hamster cells by the heavy reliance on A-NHEJ, the ability to de-repress A-NHEJ—as scored by these extrachromosomal assays—is not conserved.

## Discussion

We have utilized rAAV knockout technology to construct a powerful reagent: a series of isogenic human cell lines that are defective for genes required for the C-NHEJ-mediated branch of DNA DSB repair. We used these cell lines along with several informative reporter systems to demonstrate that wild-type human cells vastly prefer to utilize C-NHEJ over A-NHEJ for end joining reactions. Unexpectedly, the absence of the proximal C-NHEJ factor Ku, resulted in cells that still carried out robust levels of end joining, suggesting that Ku normally suppresses other end joining pathways. This model was supported by the construction of double mutant cell lines, which demonstrated that the reduction of Ku in a cell that was incapable of carrying out C-NHEJ still resulted in high levels of end joining. Thus, these studies demonstrate that Ku is the critical regulator for determining pathway choice in human somatic cells.

### Ku, the “mother” of all DSB repair inhibitors?

Ku is a heavily researched DNA repair factor and the majority of studies rightfully concentrate on some aspect of Ku's ability to positively facilitate the myriad of repair and recombination reactions that require C-NHEJ. In this and related studies, we have recently documented that Ku has an additional and hitherto underappreciated function—it is a powerful inhibitor for all the other DNA DSB repair pathways. Specifically, we have demonstrated that Ku is an essential repressor of HR-mediated aberrant telomere recombination. In the absence of Ku, the HR apparatus can apparently gain access to the telomeric ends and generate lethal telomeric shortening [43],[44]. Thus, Ku can inhibit HR specifically at telomeres. Moreover, in a study that characterized generalized rAAV-mediated gene targeting—a process that requires HR—for loci scattered throughout the genome the absence of Ku resulted in a ∼10-fold increase in correct gene targeting [42]. Importantly, the increase in correct gene targeting came at no expense to random integrations. These data strongly suggested that the reduction of Ku in human somatic cells de-repressed HR enough to facilitate much higher levels of gene targeting while simultaneously allowing other repair pathways to carry out random integrations at wild-type levels. This conclusion is completely supported by the data provided in this present study. Thus, here we have documented that in the absence of Ku, A-NHEJ is greatly up-regulated. Together, these and other [5],[37] studies have revealed that Ku can inhibit HR at telomeres, and it can inhibit HR and A-NHEJ throughout the genome. Moreover, this work and the work of Fattah *et al*. [42] make the strong prediction that the random rAAV integrations observed in Ku-deficient cells are mediated by A-NHEJ.

### Is the inhibitory activity of Ku conserved?

Although we observed the reliance on A-NHEJ in Ku-deficient hamster cell lines, we did not observe the strong de-repression of A-NHEJ activity that we had observed for human somatic cells (Figure 8A). An identical lack of de-repression using the same assay system was reported in a set of independent hamster Ku86 mutant cell lines [37] and a similar conclusion had previously been reached using extracts derived from these hamster mutants *in vitro* [16]. However, this de-repression has been observed in rodents by a number of investigators, predominately in mouse MEF or ES cell lines [15],[19],[21],[64],[71], but also in hamster cell lines [17],[18],[20]. The difference between these studies is that ours and the ones where de-repression was not observed were performed using extrachromosomal reporters and those where de-repression was observed were performed with chromosomally integrated reporter constructs. Thus, it appears in mammals as if the presence of Ku is more critical for chromosomal DSBs (which are of course the most biologically relevant substrate) and their repair than for extrachromosomal DSB repair. The basis for this distinction is not known, but it is very likely linked to Ku's ability to affect chromatin structure and the impact (direct or indirect) that this may have on repair processes. Consistent with this view, Ku separation-of-function mutations, which specifically alter Ku's heterochromatin activities, have been demonstrated to affect yeast Ku's ability to modulate telomere recombination [72]. Together, these results suggest that the difference between humans and rodents is not one of kind, but one of degree. Thus, virtually all of the loss-of-function phenotypes that have been described for human Ku can be observed in rodent Ku mutant lines, although the effects are generally—and with profound consequences—ameliorated.

### How does Ku orchestrate all this inhibition?

Many models can be envisioned for how Ku suppresses A-NHEJ. One possibility is that Ku, via direct protein∶protein interaction, sequesters a key A-NHEJ factor from performing its function. In a Ku-deficient cell, this factor would be free to facilitate A-NHEJ. A good candidate for such a putative factor exists. Thus, a bevy of independent laboratories have demonstrated that PARP-1 interacts with Ku [73]–[77]. And a PARP-1 interaction domain has been defined in the Ku70 subunit at AA243–261 [76]. This model predicts that a cell expressing a Ku70 incapable of interacting with PARP-1 (*e.g*., mutated at residues AA243–261) would phenocopy the Ku loss-of-function mutations and we are attempting to construct such a cell line. A second, and in our minds, likelier possibility, is that Ku controls A-NHEJ by regulating access to the substrate; namely, a dsDNA end. We prefer this model because not only does Ku repress A-NHEJ but it also represses HR at internal loci [42] and at telomeres [43],[44],[72]. While it is possible that Ku mediates all of this repression by physically binding to and inhibiting/sequestering a different protein or proteins for each reaction, it seems simpler if Ku simultaneously regulates all three processes by regulating access to the substrate for all of these pathways: double-stranded DNA ends. Specifically, we propose that in order to be channeled into a particular pathway (HR, A-NHEJ or C-NHEJ), that pathway's DNA binding factor (probably RAD52, PARP-1 and Ku, respectively) needs to bind onto the ends of the break and subsequently recruit their pathway's associated factors. We posit that Ku generally gets to the ends of a dsDNA break faster and/or with higher affinity than RAD52 or PARP-1 and once there it blocks their access, such that repair is fated to occur by C-NHEJ. This model is by no means novel and has been proposed by several investigators and was broached at least a decade ago [78], although it still remains largely untested. In this regard, a cell line that expressed a double-stranded DNA end binding defective Ku mutant would be predicted to be incapable of repressing either HR or A-NHEJ. Lastly, this model, in particular, could explain the differences between mice and humans. In mice, the levels of Ku/DNA-PK are much lower than they are in humans and consequently there might be a “fair fight” between Ku, RAD52 and PARP-1 over who gets to a broken end. In contrast, in human cells where the levels of Ku/DNA-PK are about 50-fold higher [11],[79], Ku has become the “bully” and essentially dominates pathway choice.

### Is there evidence for yet another sub-pathway of NHEJ?

In this study we have interrogated our mutant cell lines with two structurally similar, but fundamentally different, types of DNA ends. In one of them (*HindIII*-linearized pEGFP-Pem1-Ad2) a region of pre-existing microhomology was presented to the cell. In the other (*I-SceI*-linearized pEGFP-Pem1-Ad2 and linearized pDVG94) some processing by the cell was required to reveal the microhomology. Microhomology-mediated end joining of the *HindIII*-linearized pEGFP-Pem1-Ad2 plasmid could be detected in the wild-type parental cells (Figure 5 and Tables S1, S3, S5, S7). This perfect end joining increased in Ku heterozygotes and became the dominant reaction product in Ku- reduced/null cell lines. These results can be most simply interpreted if Ku inhibits A-NHEJ and as the level of Ku is reduced the levels of A-NHEJ reciprocally rise. The data generated using the two repair substrates that required processing suggests that this model is, however, over-simplified. Thus, as the level of Ku was reduced in the various cell lines the frequency of microhomology-mediated end joining increased with *I-SceI*-linearized pEGFP-Pem1-Ad2 and linearized pDVG94, but so did other end joining activities. This was most evident in the experiments using pDVG94 where ∼55% of the repaired plasmids in a Ku-null cell did not use microhomology to repair the plasmid (Figure 7C). Sequencing of these events and those derived from *I-SceI*-linearized pEGFP-Pem1-Ad2 did not reveal any novel repair signatures and most events looked indistinguishable from typical C-NHEJ products (Table S2). Together, these studies suggest that there may be at least one additional NHEJ pathway that is distinguishable from C-NHEJ and A-NHEJ by its lack of requirement for Ku and its lack of microhomology use, respectively. Needless to say, these products could also be accounted for by A-NHEJ if the pathway does not have an absolute requirement for microhomology. The construction of humans cell lines that are doubly defective for C-NHEJ and A-NHEJ should genetically address this issue.

### The power of rAAV-mediated human somatic cell genetics

Advances in the DNA DSB repair field have come predominately from studies on yeast and genetically modified mice. There are instances, C-NHEJ foremost among them, however, where the phenotypes of yeast and mice mutants do not accurately recapitulate the corresponding phenotypes observed in humans. Since ultimately we wish to apply what we have learned in model systems to the study of humans in the clinic, a potentially more appropriate model system is the use of human somatic cells in culture. There are, of course, attendant limitations to using human cells in culture and the requisite caution needs to be taken in extrapolating cell culture results to patients in the clinic. It is, however, also reasonable to expect that the physiology of human cells in culture may reflect more accurately the basic biochemical process of human patients than, say, rodent cell *in vivo* might. The strength of the rodent system stems predominately from the ability to make targeted alterations of individual genes using the technology of HR [80]. This technology exists for human somatic cells as well [46],[47],[81]. Overall, at least 77 different genes have been functionally inactivated in a total of 45 different human somatic cell lines {[47] and unpublished data}. To our knowledge, however, this is one of the first reports of the systematic inactivation of a large number of genes involved in a single pathway. Importantly, we have shown that it is possible to make simple knockouts, conditional knockouts and double mutant human somatic cell lines with relative ease. The general utility of rAAV-mediated gene targeting may thus be of interest for investigators working on biological problems that cannot be adequately modeled in, for example, the mouse.

## Materials and Methods

### Cell culture

The human wild-type HCT116 cell line and its derivatives were cultured in McCoy's 5A medium containing 10% fetal bovine serum, 100 U/ml penicillin, and 100 U/ml streptomycin in a humidified incubator with 5% CO_2_ at 37°C. Cell lines derived from correct gene targeting were propagated under G418 (1 mg/ml) selection. Cell lines carrying exogenous cDNA expression vectors (either XLF or DNA-PK_cs_) were grown in 2 µg/ml of puromycin.

### Cell lines

The wild-type human HCT116 cell line was obtained from the ATCC. The derivative Ku70^+/−^ [50], Ku86^+/−^ [40], Ku86^flox/−^ [44], DNA-PK_cs_^+/−^ and DNA-PK_cs_^−/−^ [51] cell lines have been described. Derivatives of Ku70^+/−^ cells treated with Ku70 RNAi (SMARTPool oligonucleotides; Dharmacon) or stably expressing shRNA vectors directed against Ku70 have also been described [42]. The XLF^+/−^ and XLF^−/−^ (Fattah *et al*., manuscript in preparation) and the LIGIV^+/−^ and LIGIV^−/−^ (Oh *et al*., manuscript in preparation) cell lines were generated by rAAV gene targeting. Similarly, compound mutant cell lines (*e.g*., Ku70^+/−^∶LIGIV^−/−^) were generated using the rAAV targeting technology described elsewhere [47].

### Treatment of Ku86^flox/−^ cells with Cre

To generate Ku86-null cells, the Ku86^flox/−^ cells were plated onto a 6-well plate at a density of 5×10^4^ cells per well and allowed to attach for 18 hr. Adenoviral infection was carried out by adding 2 ml of fresh media containing 5×10^8^ virus particles of either a control (AdCMV) or experimental (AdCre) adenoviral stock to each well [44]. After 4 days (96 hr) of incubation the cells were re-plated into 6-well plates and allowed to incubate for another 24 hr before the cells were transfected with linearized NHEJ substrates (see below). Flow cytometry was then carried out after an additional incubation for 24 hr.

### The end-joining assay, transfection, and FACS analyses

The *in vivo* end-joining reporter plasmid pEGFP-Pem1-Ad2 (Figure 1) has been described [37],[48]. Prior to transfection, the pEGFP-Pem1-Ad2 plasmid was digested with *Hind*III or *I-Sce*I (NEB) for 8 to 12 hr to generate different types of DNA ends. A pCherry plasmid (Clontech) was co-transfected with linearized pEGFP-Pem1-Ad2 as a control of transfection efficiency. The cell line under analysis was subcultured a day before transfection and was ∼60 to 70% confluent for transfection. Transfections were performed using Lipofectamine 2000 (Invitrogen) according to manufacture's instructions. Green (EGFP) and red (Cherry) fluorescence was measured by fluorescence-activated flow cytometry (FACS) 24 hr later [37]. For FACS analysis cells were harvested, washed in 1X PBS and fixed using 2% paraformaldehyde. FACS analysis was performed on a FACSCalibur instrument (BD Biosciences). For the HCT116 cell line a red-versus-green standard curve was derived with varying amount of cherry and green plasmids to avoid measurements near the plateau region. The values of repaired events are reported as a ratio of cells that were double positive for red and green fluorescence over total cells that are only positive for red fluorescence. This ratio normalizes the repair events to the transfection controls. The values for all the mutants are reported as a percent repair of wild-type cells.

### Plasmid rescue

The repaired NHEJ reporter pEGFP-Pem1-Ad2 substrates were rescued from human cells using a Qiagen mini-preparation protocol, transformed into *E. coli* (TOP10) and colonies carrying the repaired plasmids were selected on LB plates containing 30 µg/ml of kanamycin. The fidelity of NHEJ repair events was examined by digesting the plasmid DNA from individual colonies with the restriction enzyme *Hind*III prior to agarose gel electrophoresis. Precise junctional information was obtained by DNA sequencing using a variety of primers (sequences available upon request) located upstream and downstream of the Ad2 exon sequence. Those events that had not restored the original restriction site were always analyzed by sequencing. For *I-Sce*I-digested substrate, all the repair products were directly sequenced, as incompatible *I-Sce*I sites should not restore the original restriction site(s).

### Microhomology assay

The microhomology assay was performed as described [26]. In brief, 2.5 µg of *EcoRV*- (NEB) and *AfeI*- (NEB) digested plasmid pDVG94 were transfected into cells that were ∼60% confluent, in 6 well plates, using Lipofectamine 2000 (Invitrogen) according to manufacturer's instruction. The transfection efficiencies of wild-type HCT116 and the derivative mutant cell lines were determined using the plasmid pEGFP-Pem1 as described above. After transfection (48 hr), plasmid DNA was recovered using a modified Qiagen mini-preparation protocol. Repaired pDVG94 plasmid was PCR amplified using primer FM30 and a 5′-radiolabeled primer DAR5 [26]. The PCR product was digested with *BstXI* (NEB). Restriction fragments were separated by electrophoresis along with undigested PCR product in a 6% polyacrylamide gel in TBE buffer. The gel was subsequently dried and exposed to film. The bands representing the undigested (180 bp) or digested (120 bp) PCR products were quantified using ImageQuant software.

### RNA silencing

All RNA interference reagents were purchased from Dharmacon, including SmartPool siRNA pools against Ku70. Ku70 or scrambled control siRNAs (0.5 µM) were combined in a 1∶1 fashion with Dharmafect-1 reagent in a total volume of 400 µL. After a 20 min incubation at room temperature the siRNA solution was diluted to 2.0 mL with complete growth media and then added to the target cells. This procedure was repeated at least one additional time at ∼24 hr intervals.

## Supporting Information

Figure S1 The indicated cell lines were transfected with *HindIII-* (Top panels) or *I-SceI-* (Bottom panels) linearized pEGFP-Pem1-Ad2 together with a supercoiled pCherry plasmid (to monitor transfection efficiency). The number in the top right corner corresponds to the percentage of cells that turned green after 24 hr as a percentage of the cells productively transfected.
(6.55 MB TIF)

Figure S2 The data presented individually in Tables S1, S3, S5, and S7 using the *HindIII-*linearized pEGFP-Pem1-Ad2-lenearized plasmid was consolidated into 4 categories: perfect joins (dark rectangles), imperfect joins (light gray rectangles), microhomology (white rectangles), and insertions (dark gray rectangles) and is presented as the percentage of total events for each of the indicated cell lines.
(8.38 MB TIF)

Figure S3 The data presented individually in Tables S2, S4, S6, and S8 using the *I-SceI-*linearized pEGFP-Pem1-Ad2-lenearized plasmid was consolidated into 3 categories: imperfect joins (light gray rectangles), microhomology (white rectangles) and insertions (dark gray rectangles) and is presented as the percentage of total events for each of the indicated cell lines. *N.B.* Perfect joining is not possible with this substrate.
(8.70 MB TIF)

Figure S4 The data presented individually in Tables S1, S3, S5, and S7 using the *HindIII-*linearized pEGFP-Pem1-Ad2-lenearized plasmid was analyzed only for deletions. Each dot represents an individual data point and some dots overlap. The mean (dark rectangle) and the median (gray rectangle) are shown for each of the indicated cell lines.
(8.41 MB TIF)

Figure S5 The data presented individually in Tables S2, S4, S6, and S8 using the *I-SceI-*linearized pEGFP-Pem1-Ad2-lenearized plasmid was analyzed only for deletions. Each dot represents an individual data point and some dots overlap. The mean (dark rectangle) and the median (gray rectangle) are shown for each of the indicated cell lines.
(8.55 MB TIF)

Table S1 Sequence analysis for *HindIII*-linearized pEGFP-Pem1-Ad2 plasmids recovered from Ku-deficient cells.
(10.54 MB TIF)

Table S2 Sequence analysis for I-SceI-linearized pEGFP-Pem1-Ad2 plasmids recovered from Ku-deficient cells.
(10.06 MB TIF)

Table S3 Sequence analysis for *HindIII-*linearized pEGFP-Pem1-Ad2 plasmids recovered from DNA-PK_CS_-deficient cells.
(7.79 MB TIF)

Table S4 Sequence analysis for *I-SceI-*linearized pEGFP-Pem1-Ad2 plasmids recovered from DNA-PK_CS_-deficient cells.
(9.39 MB TIF)

Table S5 Sequence analysis for *HindIII-*linearized pEGFP-Pem1-Ad2 plasmids recovered from XLF-deficient cells.
(7.26 MB TIF)

Table S6 Sequence analysis for *I-SceI-*linearized pEGFP-Pem1-Ad2 plasmids recovered from XLF-deficient cells.
(8.34 MB TIF)

Table S7 Sequence analysis for *HindIII-*linearized pEGFP-Pem1-Ad2 plasmids recovered from LIGIV-deficient cells.
(7.31 MB TIF)

Table S8 Sequence analysis for *I-SceI-*linearized pEGFP-Pem1-Ad2 plasmids recovered from LIGIV-deficient cells.
(8.11 MB TIF)
