# Supplementary figures and images for: Ku Regulates the Non-Homologous End Joining Pathway Choice of DNA Double-Strand Break Repair in Human Somatic Cells
Source: PLoS Genet. 2010 Feb 26;6(2):e1000855. doi: 10.1371/journal.pgen.1000855 (PMC2829059; doi:10.1371/journal.pgen.1000855)

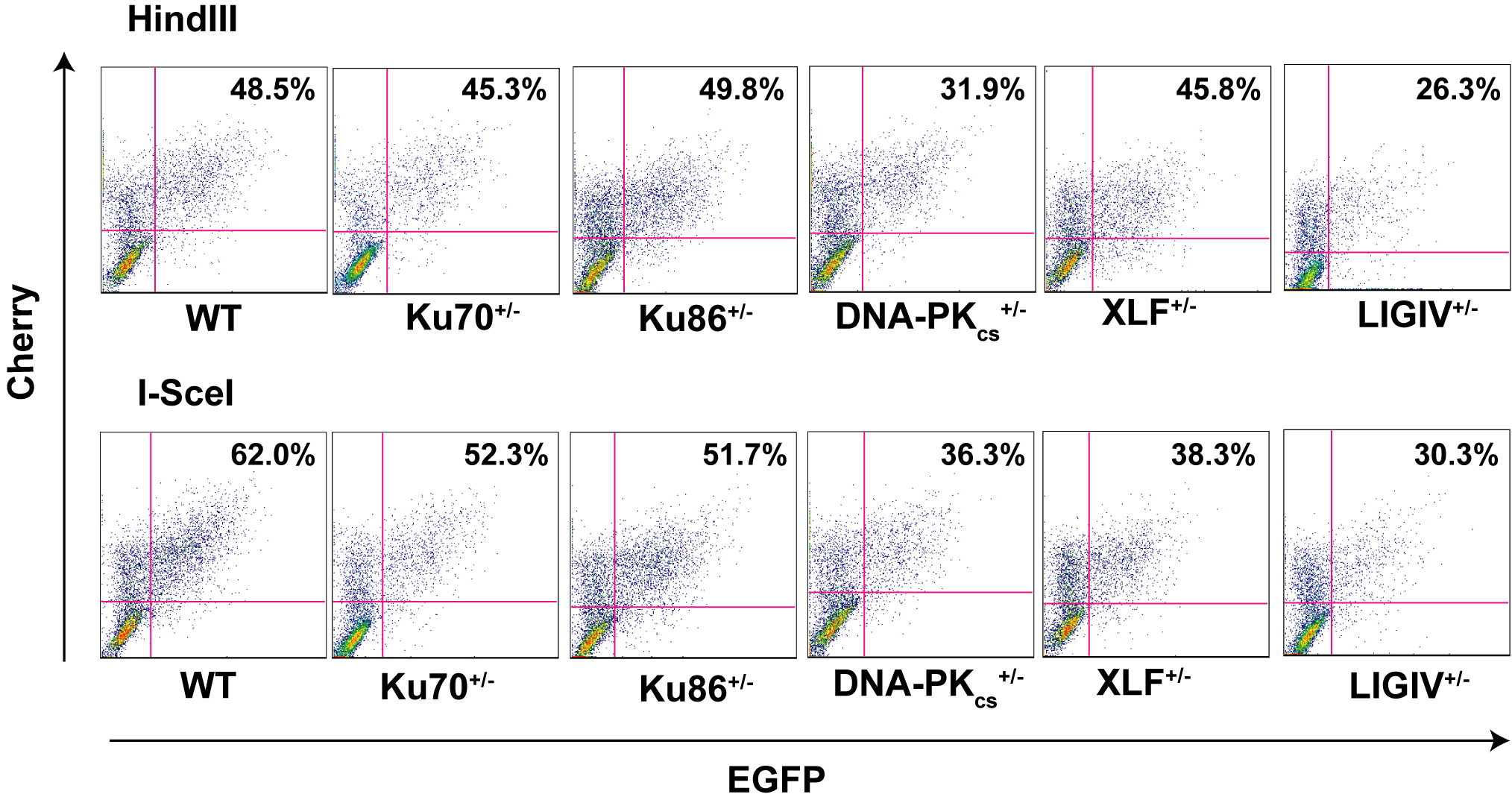

Supplement: Figure S1 — The indicated cell lines were transfected with HindIII- (Top panels) or I-SceI- (Bottom panels) linearized pEGFP-Pem1-Ad2 together with a supercoiled pCherry plasmid (to monitor transfection efficiency). The number in the top right corner corresponds to the percentage of cells that turned green after 24 hr as a percentage of the cells productively transfected. (6.55 MB TIF) [file pgen.1000855.s001.tif]

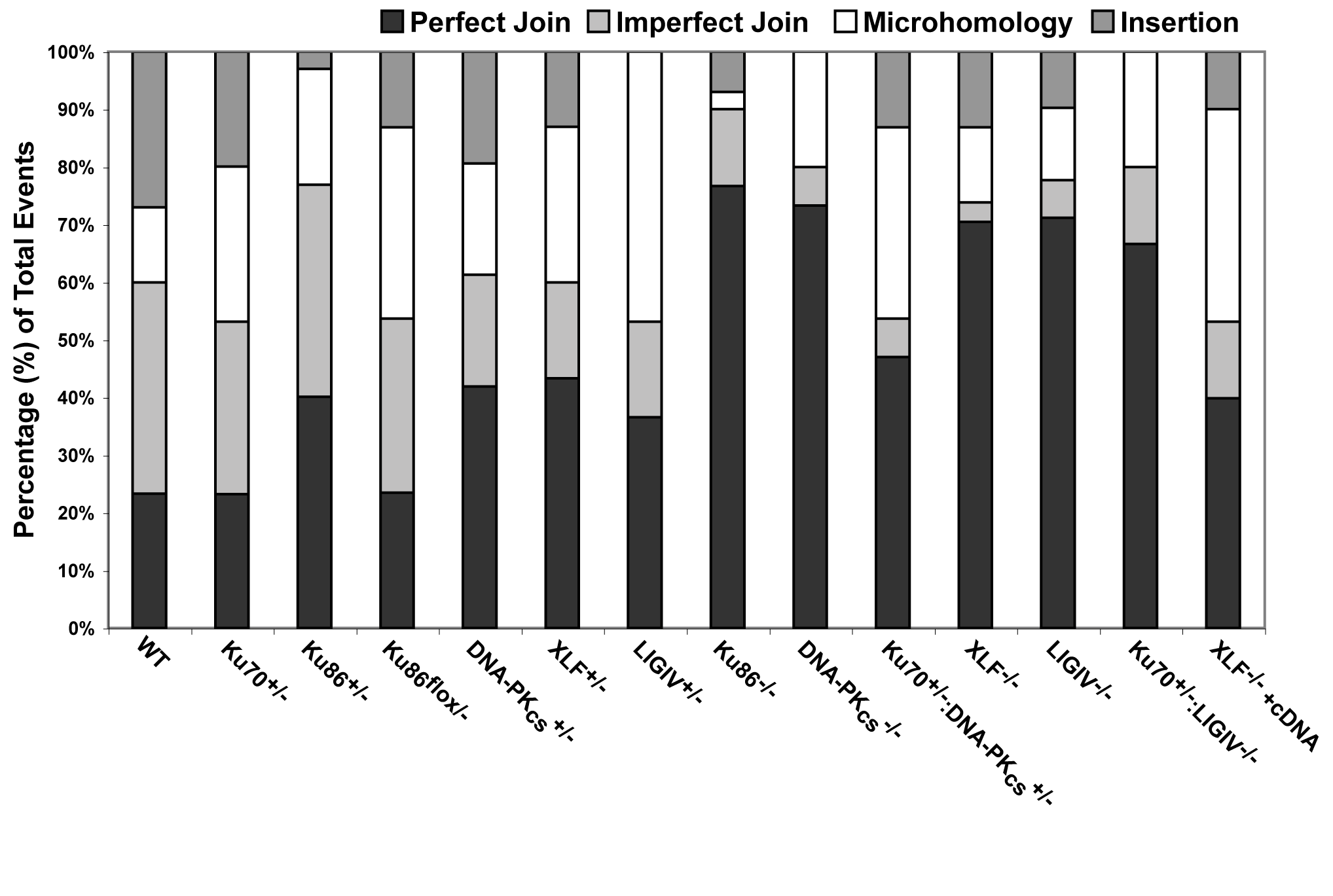

Supplement: Figure S2 — The data presented individually in Tables S1, S3, S5, and S7 using the HindIII-linearized pEGFP-Pem1-Ad2-lenearized plasmid was consolidated into 4 categories: perfect joins (dark rectangles), imperfect joins (light gray rectangles), microhomology (white rectangles), and insertions (dark gray rectangles) and is presented as the percentage of total events for each of the indicated cell lines. (8.38 MB TIF) [file pgen.1000855.s002.tif]

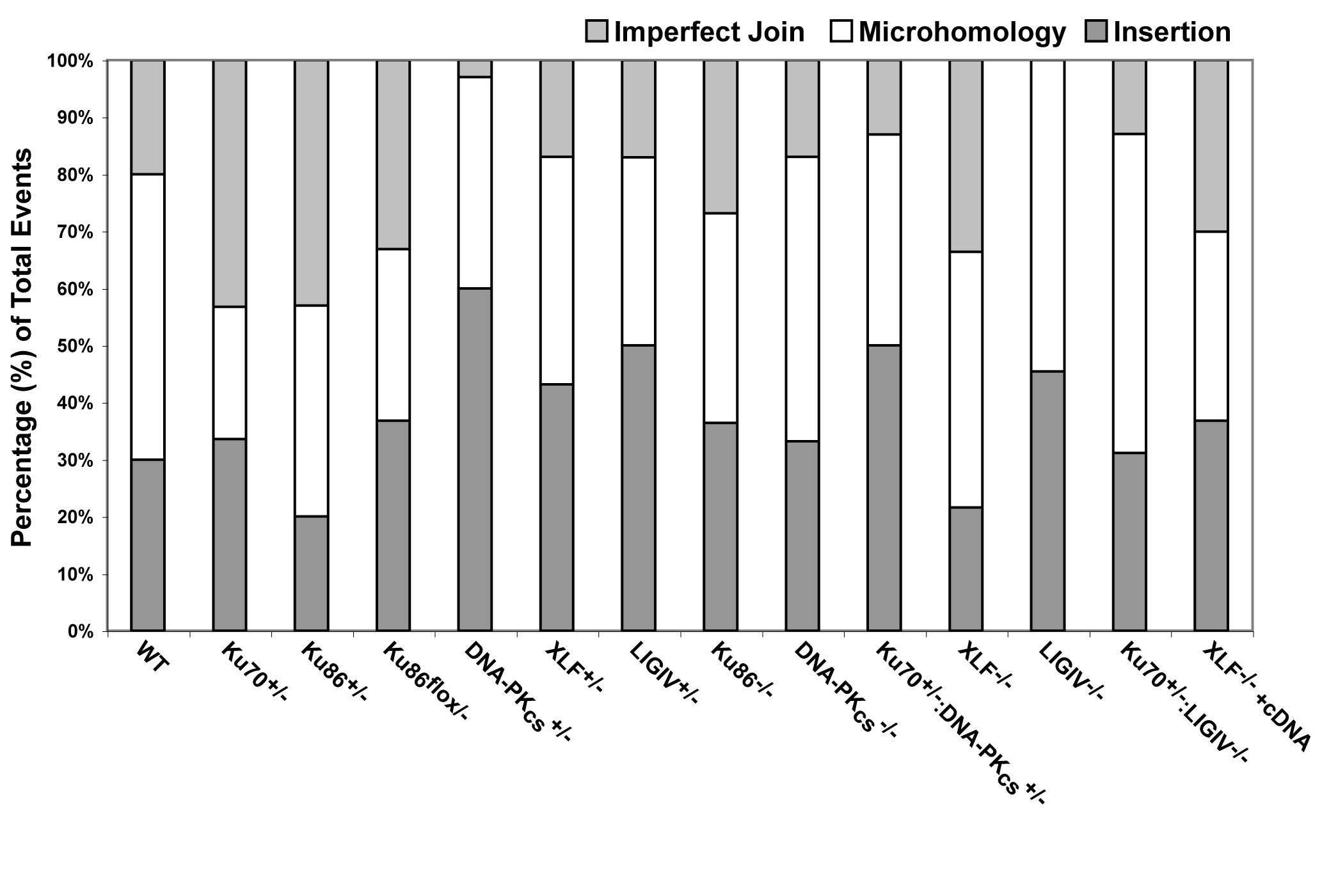

Supplement: Figure S3 — The data presented individually in Tables S2, S4, S6, and S8 using the I-SceI-linearized pEGFP-Pem1-Ad2-lenearized plasmid was consolidated into 3 categories: imperfect joins (light gray rectangles), microhomology (white rectangles) and insertions (dark gray rectangles) and is presented as the percentage of total events for each of the indicated cell lines. N.B. Perfect joining is not possible with this substrate. (8.70 MB TIF) [file pgen.1000855.s003.tif]

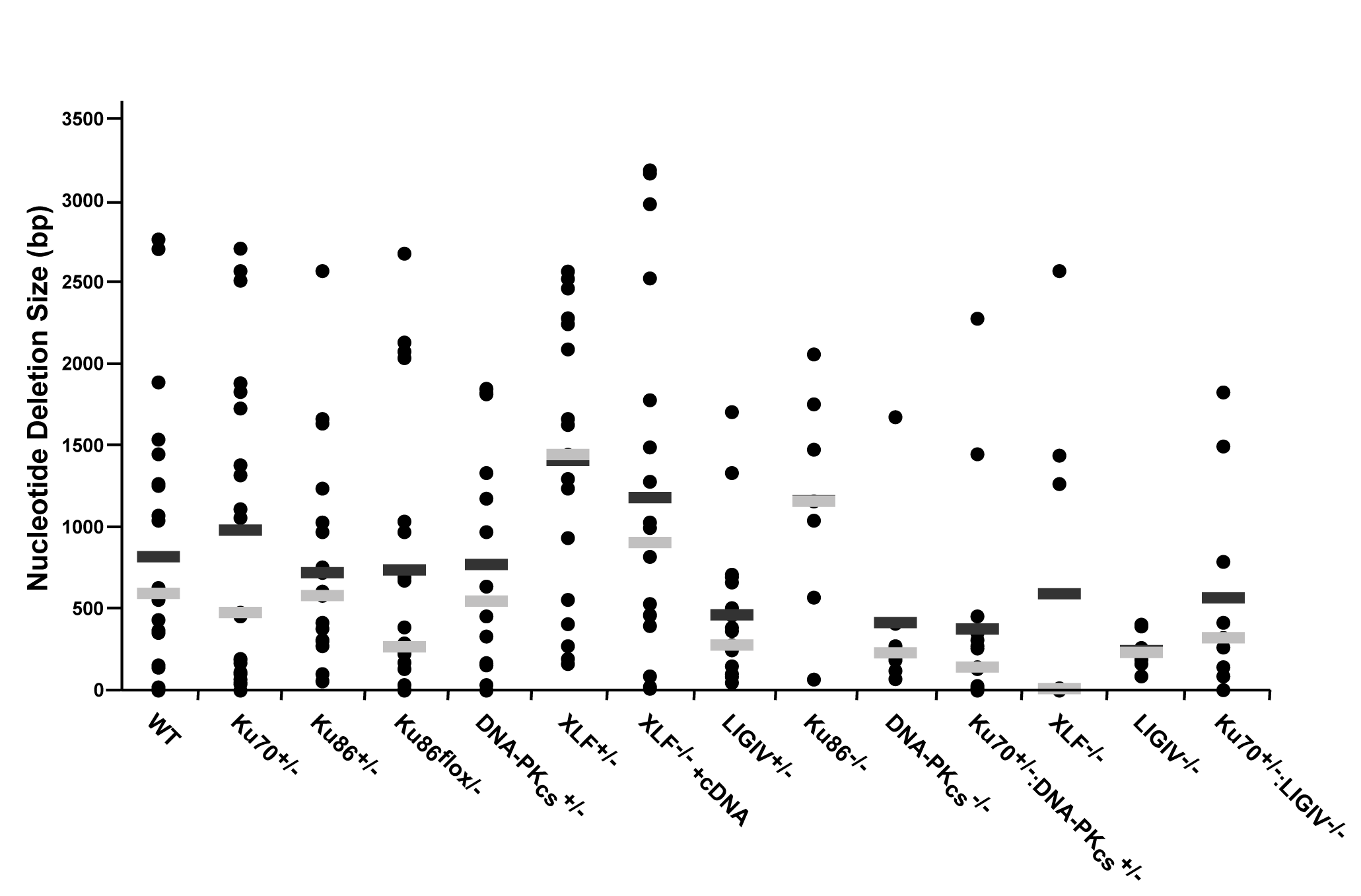

Supplement: Figure S4 — The data presented individually in Tables S1, S3, S5, and S7 using the HindIII-linearized pEGFP-Pem1-Ad2-lenearized plasmid was analyzed only for deletions. Each dot represents an individual data point and some dots overlap. The mean (dark rectangle) and the median (gray rectangle) are shown for each of the indicated cell lines. (8.41 MB TIF) [file pgen.1000855.s004.tif]

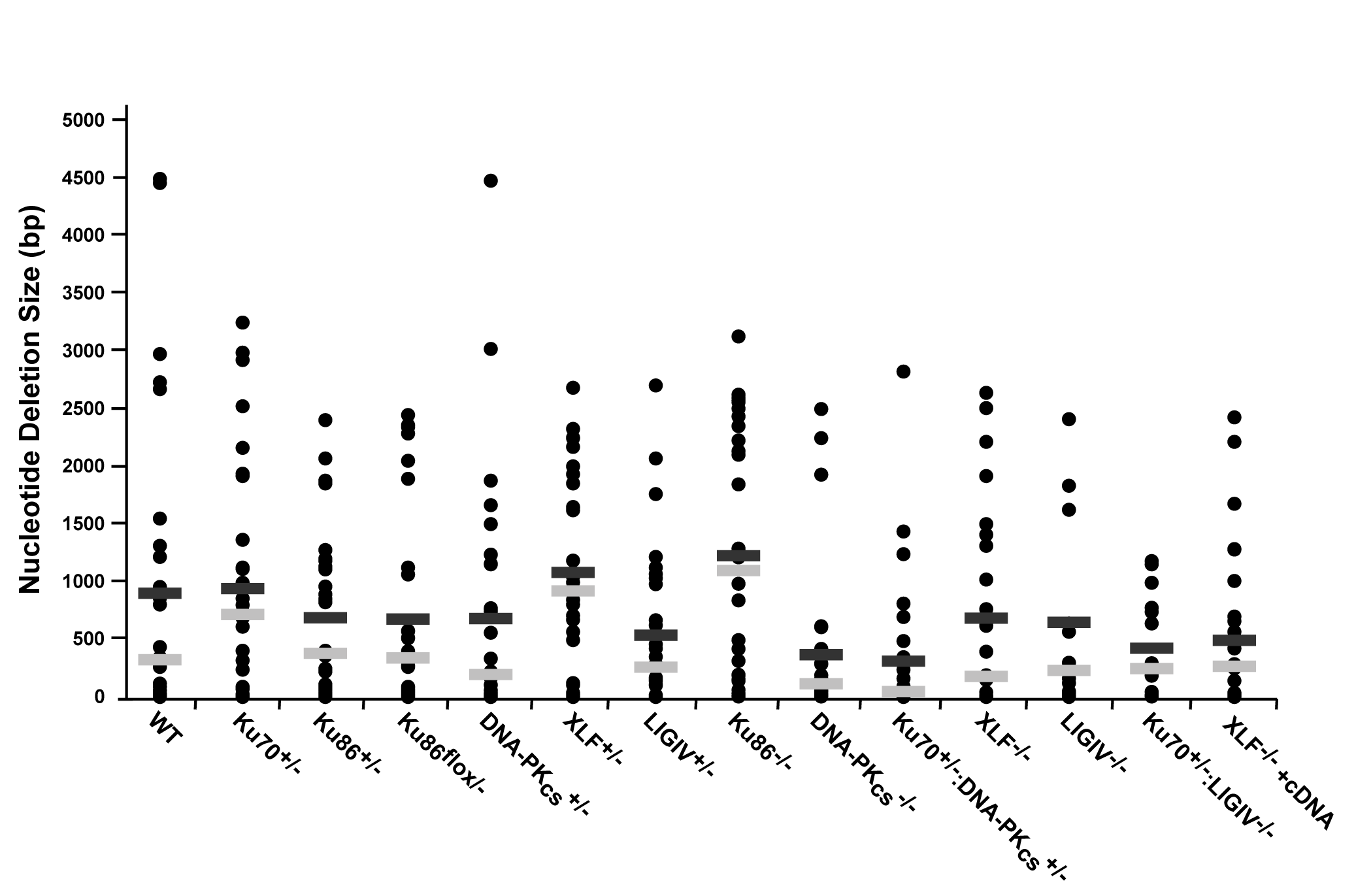

Supplement: Figure S5 — The data presented individually in Tables S2, S4, S6, and S8 using the I-SceI-linearized pEGFP-Pem1-Ad2-lenearized plasmid was analyzed only for deletions. Each dot represents an individual data point and some dots overlap. The mean (dark rectangle) and the median (gray rectangle) are shown for each of the indicated cell lines. (8.55 MB TIF) [file pgen.1000855.s005.tif]

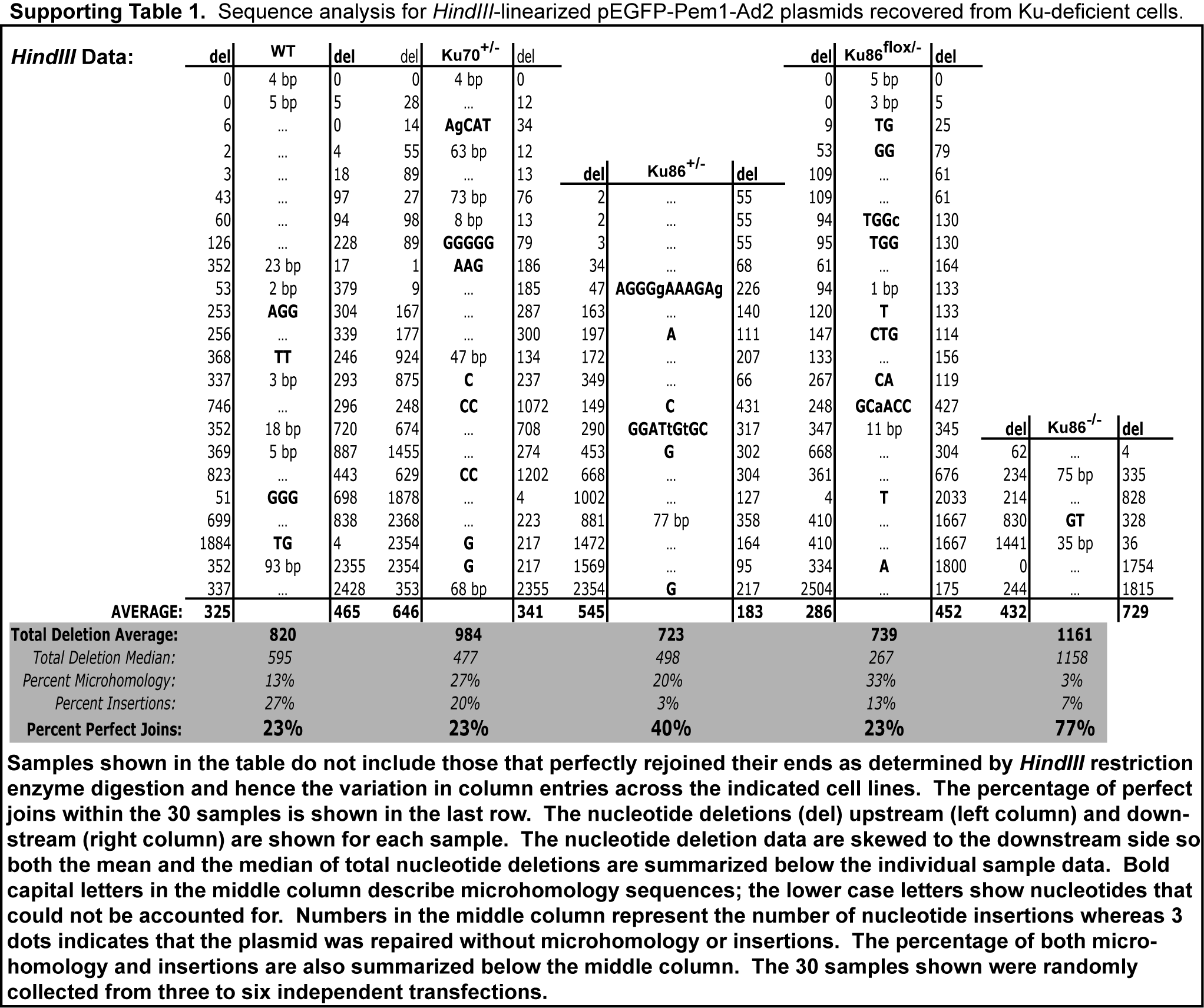

Supplement: Table S1 — Sequence analysis for HindIII-linearized pEGFP-Pem1-Ad2 plasmids recovered from Ku-deficient cells. (10.54 MB TIF) [file pgen.1000855.s006.tif]

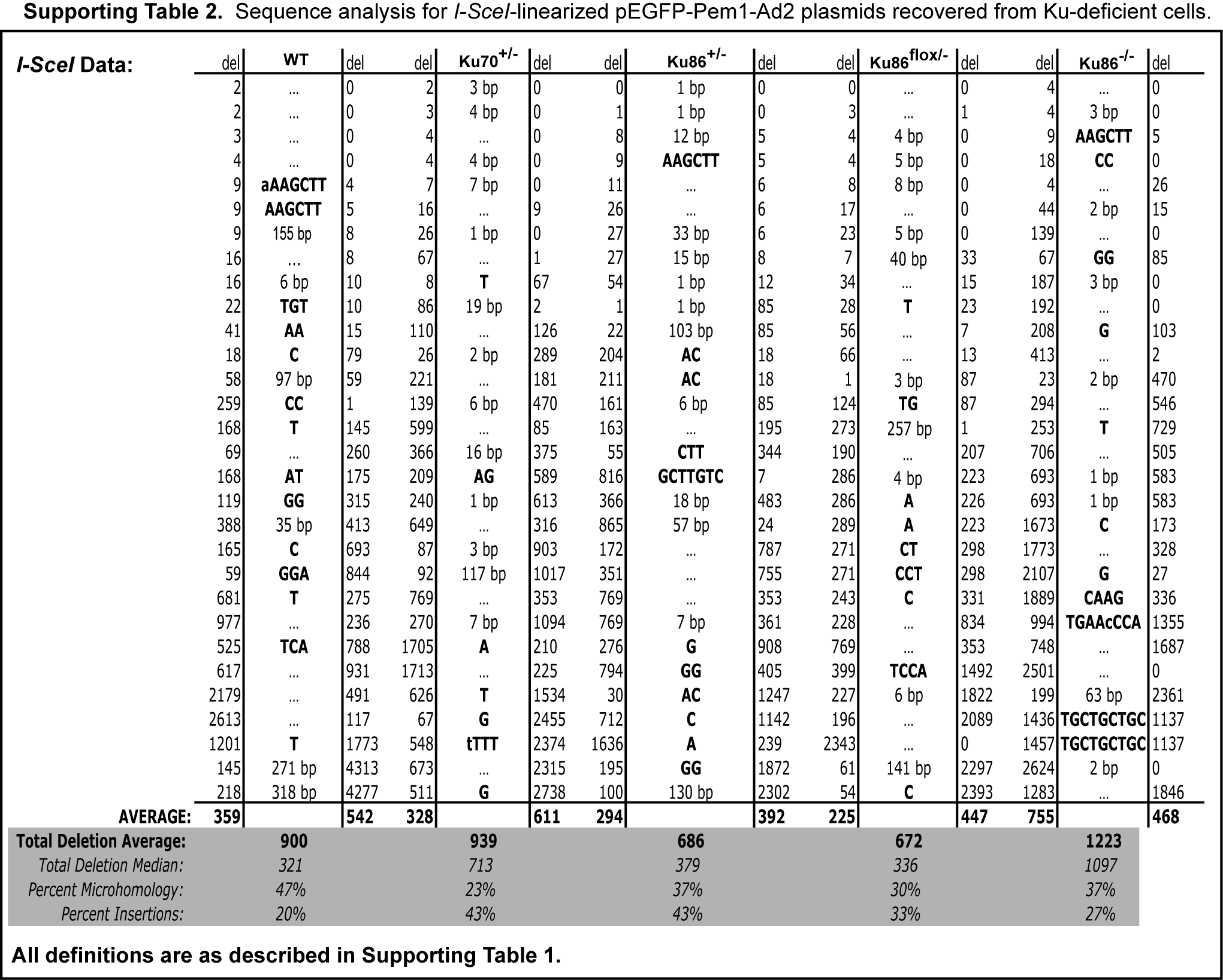

Supplement: Table S2 — Sequence analysis for I-SceI-linearized pEGFP-Pem1-Ad2 plasmids recovered from Ku-deficient cells. (10.06 MB TIF) [file pgen.1000855.s007.tif]

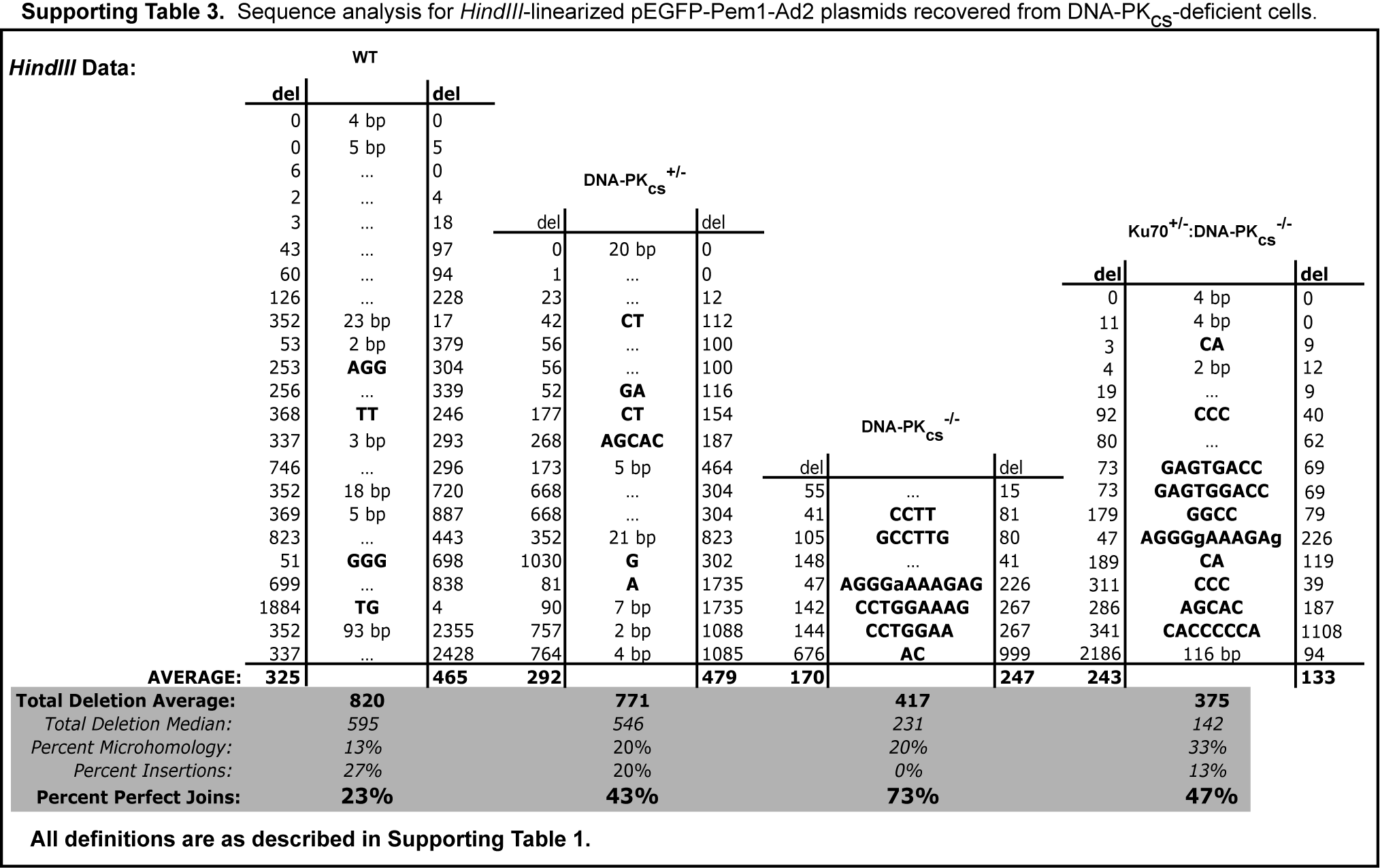

Supplement: Table S3 — Sequence analysis for HindIII-linearized pEGFP-Pem1-Ad2 plasmids recovered from DNA-PKCS-deficient cells. (7.79 MB TIF) [file pgen.1000855.s008.tif]

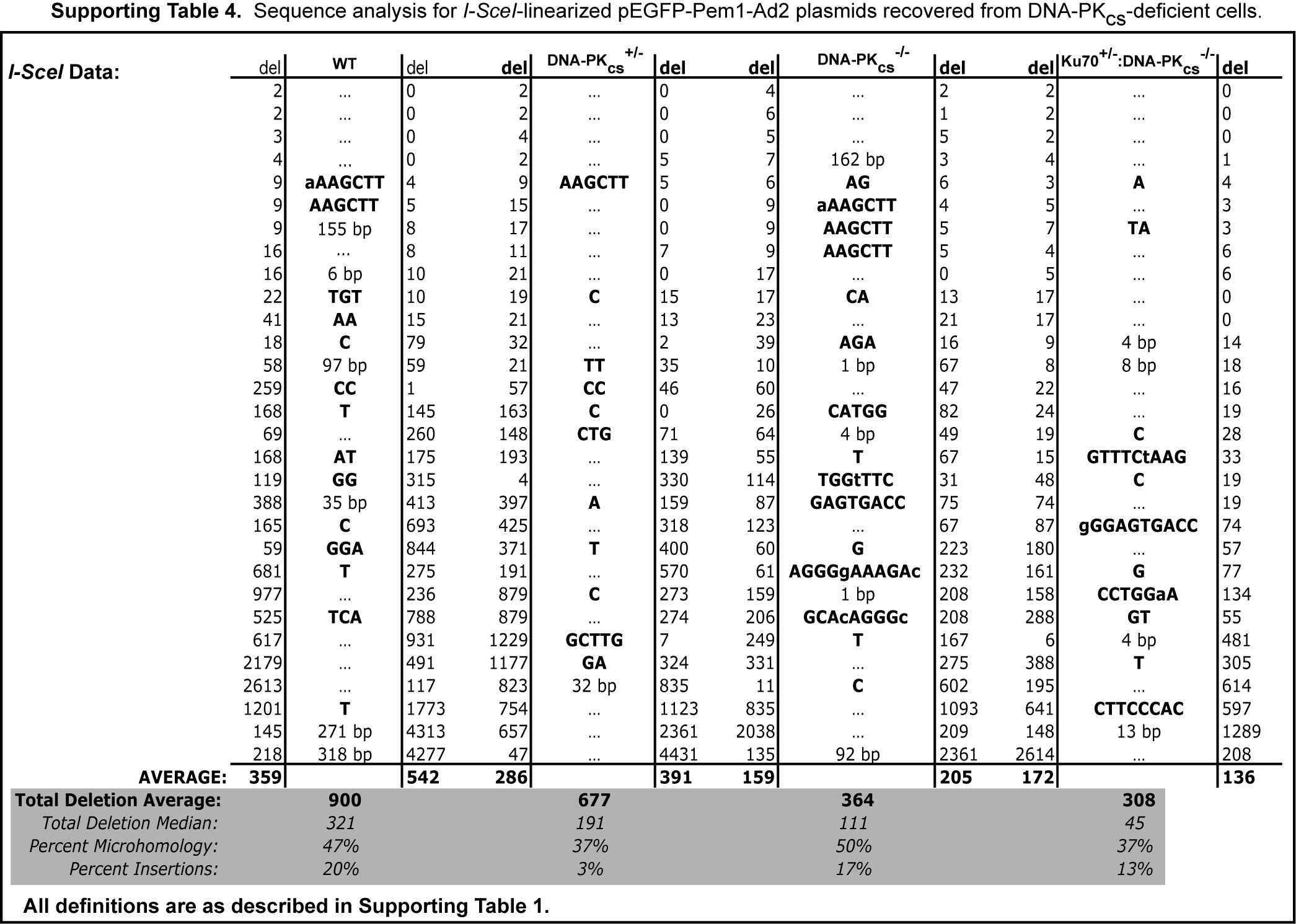

Supplement: Table S4 — Sequence analysis for I-SceI-linearized pEGFP-Pem1-Ad2 plasmids recovered from DNA-PKCS-deficient cells. (9.39 MB TIF) [file pgen.1000855.s009.tif]

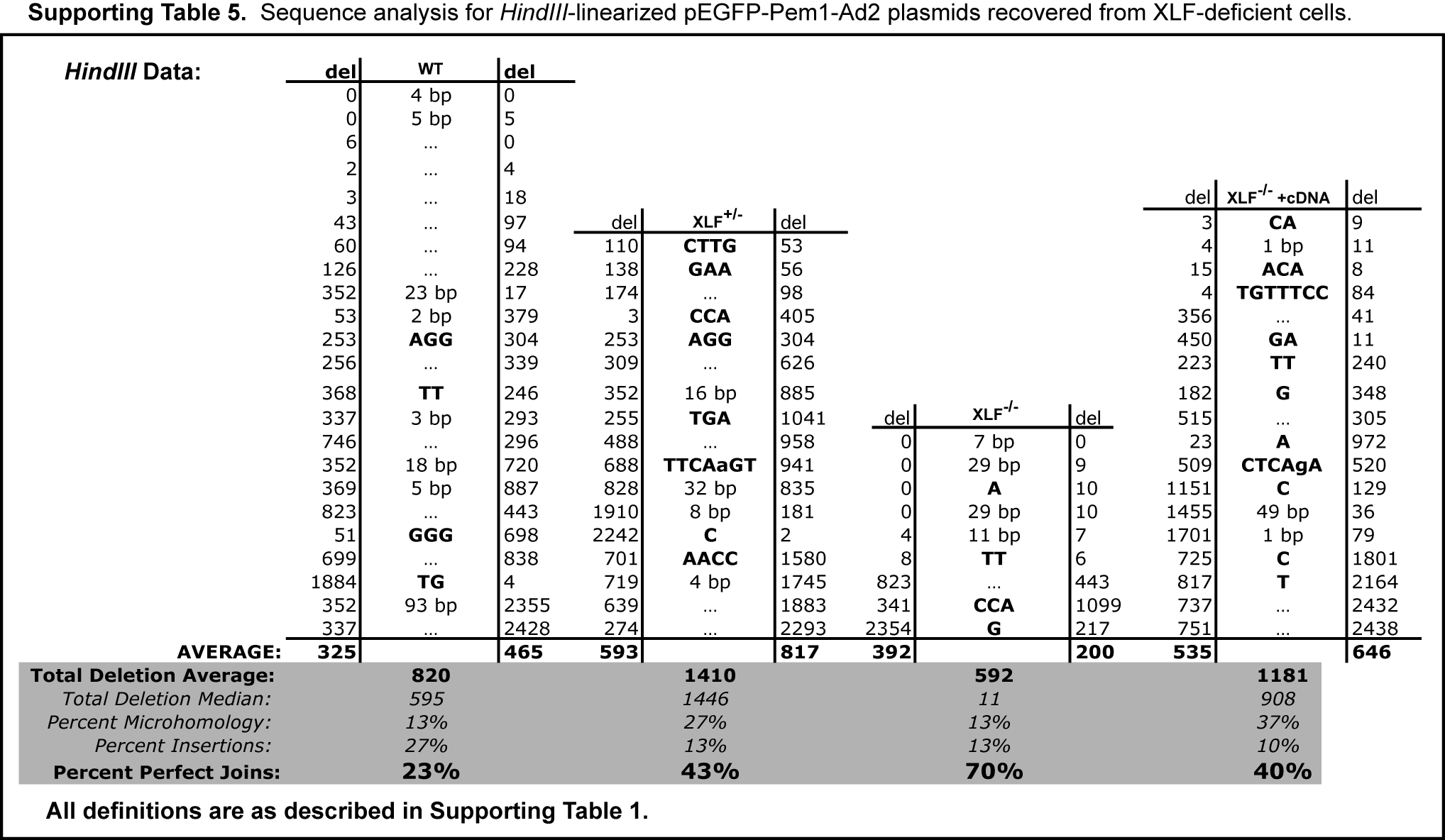

Supplement: Table S5 — Sequence analysis for HindIII-linearized pEGFP-Pem1-Ad2 plasmids recovered from XLF-deficient cells. (7.26 MB TIF) [file pgen.1000855.s010.tif]

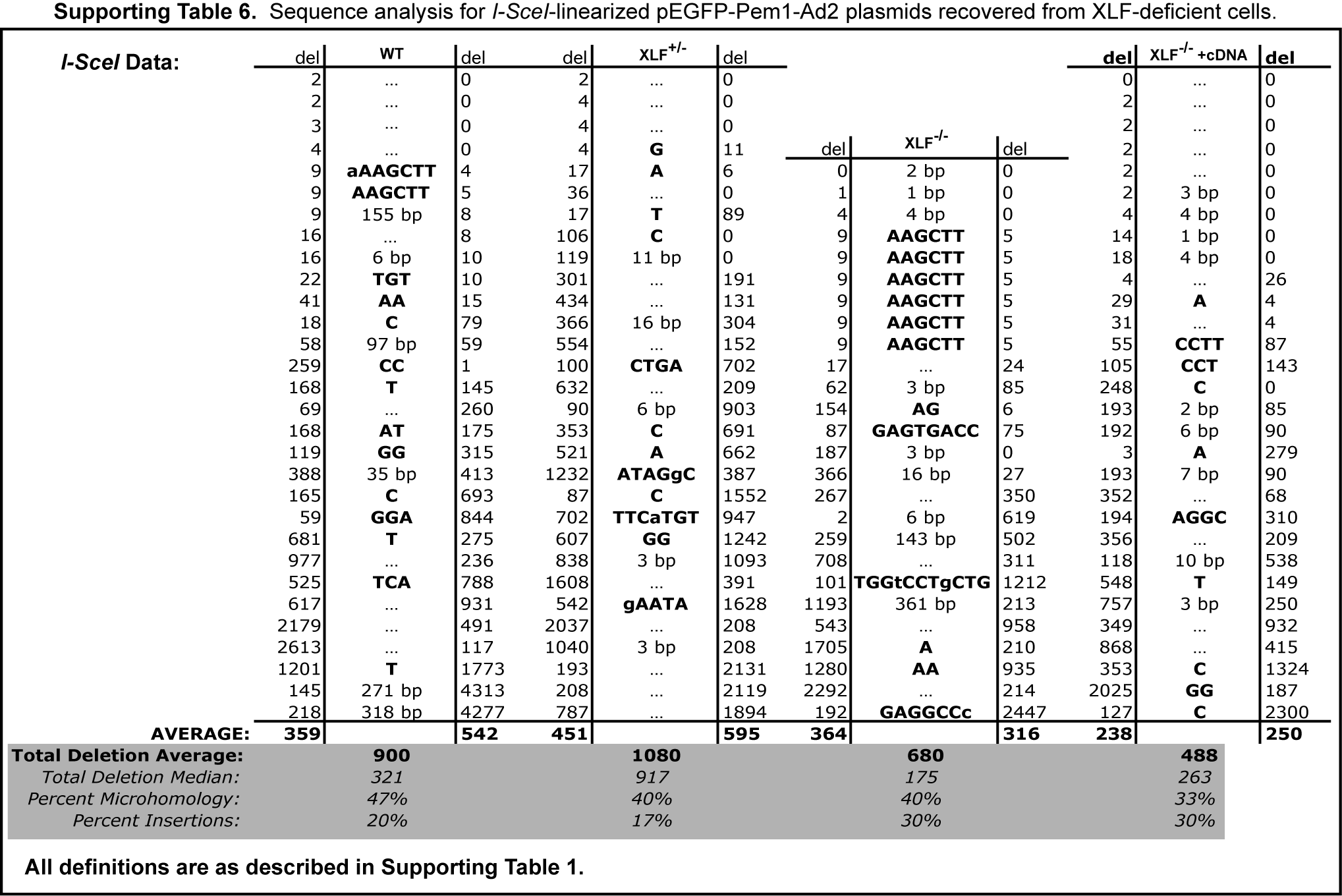

Supplement: Table S6 — Sequence analysis for I-SceI-linearized pEGFP-Pem1-Ad2 plasmids recovered from XLF-deficient cells. (8.34 MB TIF) [file pgen.1000855.s011.tif]

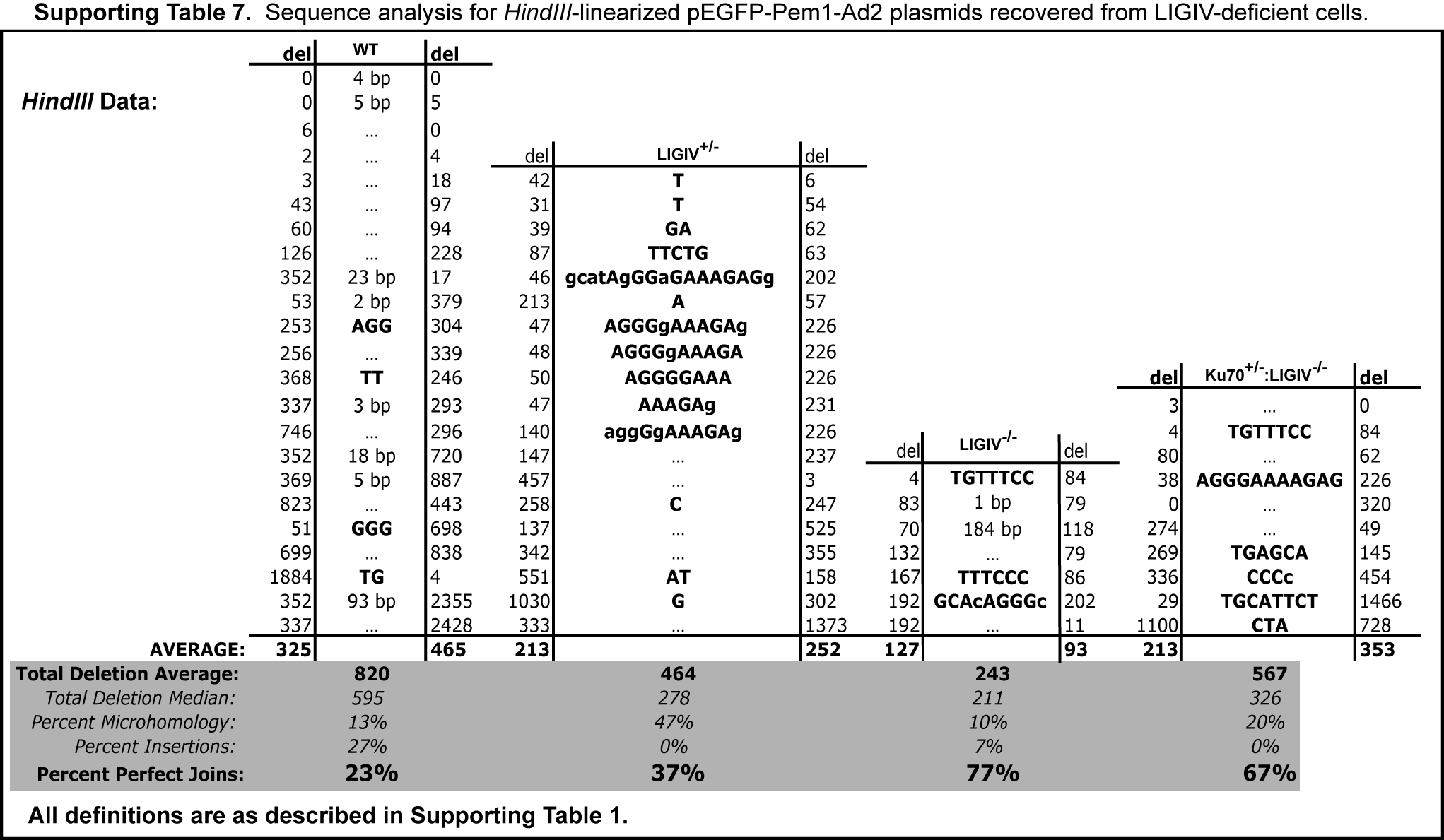

Supplement: Table S7 — Sequence analysis for HindIII-linearized pEGFP-Pem1-Ad2 plasmids recovered from LIGIV-deficient cells. (7.31 MB TIF) [file pgen.1000855.s012.tif]

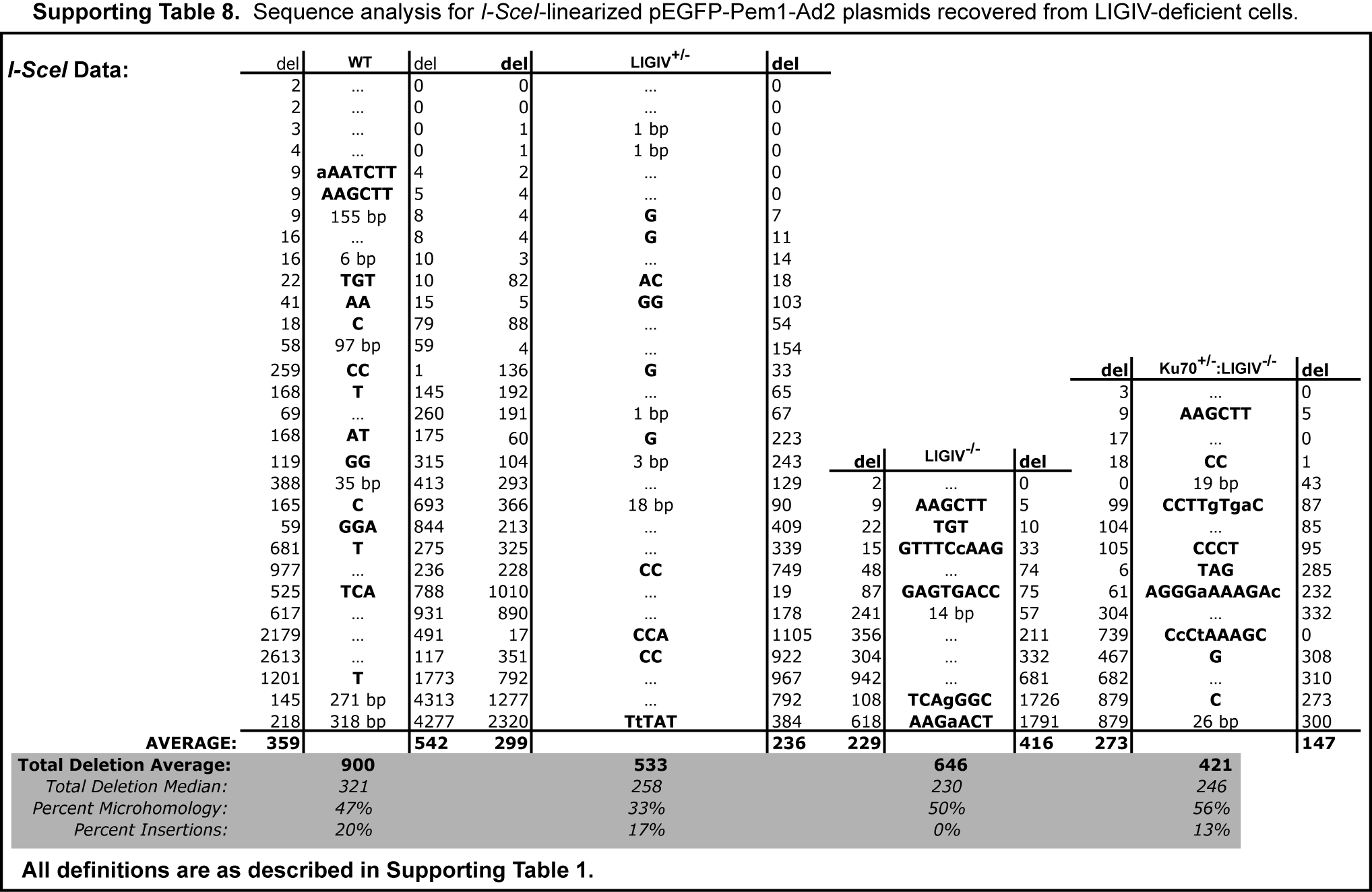

Supplement: Table S8 — Sequence analysis for I-SceI-linearized pEGFP-Pem1-Ad2 plasmids recovered from LIGIV-deficient cells. (8.11 MB TIF) [file pgen.1000855.s013.tif]
